# Resolving the genetic paradox of invasions: Preadapted genomes and postintroduction hybridization of bigheaded carps in the Mississippi River Basin

**DOI:** 10.1111/eva.12863

**Published:** 2019-09-12

**Authors:** Jun Wang, Sarah Gaughan, James T. Lamer, Cao Deng, Wanting Hu, Michael Wachholtz, Shishang Qin, Hu Nie, Xiaolin Liao, Qufei Ling, Weitao Li, Lifeng Zhu, Louis Bernatchez, Chenghui Wang, Guoqing Lu

**Affiliations:** ^1^ Department of Biology University of Nebraska at Omaha Omaha USA; ^2^ Key Laboratory of Freshwater Fisheries Germplasm Resources Ministry of Agriculture National Demonstration Center for Experimental Fisheries Science Education/Shanghai Engineering Research Center of Aquaculture Shanghai Ocean University Shanghai China; ^3^ Department of Biological Sciences Western Illinois University Macomb IL USA; ^4^ DNA Stories Bioinformatics Center Chengdu China; ^5^ College of Life of Sciences Nanjing Normal University Nanjing China; ^6^ Institute of Hydroecology Ministry of Water Resources & Chinese Academy of Sciences Wuhan China; ^7^ Aquaculture Institute School of Biology and Basic Medical Sciences Soochow University Suzhou China; ^8^ IBIS (Institut de Biologie Intégrative et des Systèmes) Université Laval Québec QC Canada

**Keywords:** bigheaded carps or Asian carp, cross experiment, genetic paradox of invasions, genome sequencing, interspecific hybridization

## Abstract

The genetic paradox of biological invasions is complex and multifaceted. In particular, the relative role of disparate propagule sources and genetic adaptation through postintroduction hybridization has remained largely unexplored. To add resolution to this paradox, we investigate the genetic architecture responsible for the invasion of two invasive Asian carp species, bighead carp (*Hypophthalmichthys nobilis*) and silver carp (*H. molitrix*) (bigheaded carps) that experience extensive hybridization in the Mississippi River Basin (MRB). We sequenced the genomes of bighead and silver carps (~1.08G bp and ~1.15G bp, respectively) and their hybrids collected from the MRB. We found moderate‐to‐high heterozygosity in bighead (0.0021) and silver (0.0036) carps, detected significantly higher dN/dS ratios of single‐copy orthologous genes in bigheaded carps versus 10 other species of fish, and identified genes in both species potentially associated with environmental adaptation and other invasion‐related traits. Additionally, we observed a high genomic similarity (96.3% in all syntenic blocks) between bighead and silver carps and over 90% embryonic viability in their experimentally induced hybrids. Our results suggest intrinsic genomic features of bigheaded carps, likely associated with life history traits that presumably evolved within their native ranges, might have facilitated their initial establishment of invasion, whereas *ex-situ* interspecific hybridization between the carps might have promoted their range expansion. This study reveals an alternative mechanism that could resolve one of the genetic paradoxes in biological invasions and provides invaluable genomic resources for applied research involving bigheaded carps.

## 1 INTRODUCTION

Introduced species can experience population bottlenecks after introduction that can reduce fitness and evolutionary potential; however, they are often able to successfully establish in introduced regions and become invasive despite this obstacle. This genetic paradox has attracted invasion biologists for decades (Allendorf & Lundquist, 2003; Estoup et al., 2016; Kolbe et al., 2004). Several mechanisms have been proposed to explain the mechanisms contributing to their invasiveness, including rapid adaptive evolution in introduced environments (Nei, Maruyama, & Chakraborty, 1975; Perez, Nirchio, Alfonsi, & Munoz, 2006; Phillips, Brown, Webb, & Shine, 2006; Vandepitte et al., 2014), multiple introductions and genetic admixture of previously isolated populations (Dlugosch & Parker, 2008; Facon, Pointier, Jarne, Sarda, & David, 2008; Hahn & Rieseberg, 2017; Kolbe et al., 2004), interspecific hybridization (Ellstrand & Schierenbeck, 2000; Mallet, 2005; Mesgaran et al., 2016), and others (Guerreiro & Fontdevila, 2011; Hoffmann & Rieseberg, 2008; Kirkpatrick & Barrett, 2015; Pandit, White, & Pocock, 2014; Prevosti et al., 1988). Many successful invaders develop life history traits in their native regions that allow the introduced populations to excel under a wide range of conditions, which supports the preintroduction adaption hypothesis (Baker & Stebbins, 1965; Kolar & Lodge, 2001). Consequently, the intrinsic genetic features that are linked to invasion‐related traits likely play an indispensable role in successful invasions, particularly at the initial establishment stage.

Bighead carp (*Hypophthalmichthys nobilis*) and silver carp (*H. molitrix*) (together, bigheaded carps or Asian carp) belong to the family Cyprinidae and are among the most cultured species in East Asia and some European countries due to their superior filter feeding, rapid growth, and high fecundity (Li, Wu, Wang, Chou, & Chen, 1990). Bigheaded carps have been widely introduced into over 70 countries and established in some 20 countries (Kolar, Chapman, & Courtenay, 2007). Both species were initially introduced into the United States (US) in the early 1970s, escaped from confinement, detected in natural waterways in the early 1980s, and have since become extremely abundant in the Mississippi River Basin (MRB; Chick & Pegg, 2001). These invasive carps outcompete indigenous species and may have dramatic negative impacts on local fisheries (Chick & Pegg, 2001; Kolar et al., 2007). Genomic approaches are powerful tools to understand the mechanisms underlying biological invasions (Chown et al., 2015). Here, we sequence the genomes of bighead and silver carps sampled from the MRB, identify genomic features such as heterozygosity and genes under selection, and discuss the possible link between intrinsic genomic features and invasion success in bigheaded carps.

Hybridization has long been hypothesized as a stimulus to biological invasions, with evidence primarily from plant systems (Baker & Stebbins, 1965; Ellstrand & Schierenbeck, 2000). Only a few vertebrate animal examples have demonstrated such a link (Hovick & Whitney, 2014), and even fewer cases exist between two introduced species hybridizing to facilitate invasion success (Haynes et al., 2012). Bighead and silver carp, albeit sympatric, are reproductively isolated within their native regions, and their hybrids are rarely found in the wild (Lamer et al., 2015). However, extensive introgressive hybridization between bigheaded carps has been reported in the MRB (Lamer et al., 2015). In the MRB, some F_1_ hybrids were observed to exhibit morphologic deformations (e.g., twisted gill rakers; Lamer, Dolan, Petersen, Chick, & Epifanio, 2010) and exhibit decreased body condition (Lamer, Ruebush, & McClelland, 2019), suggesting that the F_1_ hybrids of bigheaded carps may have lower fitness and undergo postzygotic constraints compared to their parental species (Kolar et al., 2007). However, early‐generation hybrids are more likely to disperse and are more abundant at the invasion fronts (Coulter, Brey, Lamer, Whitledge, & Garvey, 2019) that could increase population sizes and hence counter founder effects (Drake, 2006). Moreover, genetic introgression may result in heterogenotypes with potentially higher fitness and genetic resiliency, and therefore accelerate natural selection and promote invasion success (Facon, Jarne, Pointier, & David, 2005; Vila & D'Antonio, 1998). In this study, we assess the potential role of interspecific hybridization between bighead and silver carps in their successful invasions in North America by conducting comparative studies of genomes and embryonic development in pure and hybrid bigheaded carps.

## 2 MATERIALS AND METHODS

### 2.1 Ethics statement

This study was approved by the Institutional Animal Care and Use Committees (IACUC) of Western Illinois University (IL, USA). All sampling procedures complied with the guidelines of IACUC on the care and use of animals for scientific purposes.

### 2.2 Sampling

Bighead carp and silver carp samples were collected from the Marseilles Reach of the Illinois River (Morris, IL) in the MRB. We initially collected two bighead carp (one male and one female), two silver carp (one male and one female), and four reciprocal hybrid samples classified by morphological characters (Kolar et al., 2007). Further genetic screening using 57 nuclear and one mitochondrial species‐diagnostic SNPs (Lamer et al., 2015) identified one bighead carp (female) and one silver carp (male) as hybrids. Consequently, samples of one pure bighead carp, one pure silver carp, and two F_1_ hybrids were used for sequencing (Table S1). Muscle tissue of these samples (300–400 mg) was biopsied using disposable, 8‐mm surgical biopsy punches to avoid contamination from fluids of other captured fish. The tissue samples were then transported back to the laboratory on dry ice for DNA extraction.

### 2.3 Genome sequencing

DNA extraction was conducted using the Agencourt DNAdvance genomic DNA extraction kit (Beckman Coulter) according to the manufacturer's instructions. DNA extracted from bighead carp and silver carp samples was used for the construction of 170 and 450 bp short paired‐end and 2 and 5 kb large mate‐paired libraries for each species (Table S1). Two libraries (170 bp paired‐end, 2 kb mate‐paired) were constructed for each of the two F_1_ hybrids. All sequencing libraries were constructed using the standard protocol provided by Illumina. Paired‐end sequencing was performed using the Illumina HiSeq 2000 system by BGI‐Hong Kong (Table S1). The PacBio data were generated following the Pacific Biosciences (PacBio)‐recommended protocols. The library preparation followed their 10 kb Template Preparation and Sequencing protocol (PacBio: P/N 100‐152‐400‐04). Sequencing was performed by the Laboratory of Biotechnology and Bioanalysis at Washington State University (Table S1).

### 2.4 Genome assembly

Sequencing adaptors and low‐quality reads were filtered out before *de novo* assembly. A two‐step strategy was used for *de novo* genome assembly. First, Illumina reads from BGI were assembled into contigs and scaffolds with SOAPdenovo2 (Luo et al., 2012) with *K* = 35, 37, 39, 41, and 43. The gaps were then closed using PBJelly software with corrected PacBio reads (English et al., 2012). PBJelly is a pipeline for improving genome assemblies using PacBio reads (English et al., 2012), and all steps (setup, mapping, support, extraction, assembly, and output) were run with default parameters.

### 2.5 Genome annotation

#### 2.5.1 Repeated sequences

*De novo* detection of repeated sequences (repeats) in the genomes of bighead and silver carps was carried out by running RepeatModeler and RepeatMasker (Smit, Hubley, & Green, 1996). The species‐specific *de novo* repeat libraries were constructed by RepeatModeler (Smit et al., 1996) with default parameters. The consensus sequences in *de novo* repeat libraries and their classification information were used to run RepeatMasker on the assembled scaffolds, followed by further tandem repeats identification using TRF (Benson, 1999). To compare DNA repeats in the genomics of bigheaded carps and other species of fish, we used the same pipeline to analyze zebrafish (Ensembl 78), common carp (Ensembl 78), and cavefish (Ensembl 78) genomes.

#### 2.5.2 Genes and functions

*De novo* and sequence homology‐based methods were used for gene prediction. For *de novo* gene prediction, SNAP (Korf, 2004), GeneMark‐ET (Tang, Lomsadze, & Borodovsky, 2015), and Augustus (Sommerfeld, Lingner, Stanke, Morgenstern, & Richter, 2009) were used to predict genes on genome sequences with transposable elements masked. The high‐quality dataset for training these *ab initio* gene predictors was generated by PASA (Haas et al., 2003). For sequence homology‐based gene prediction, protein sequences from Swiss‐Prot vertebrates database and four model organisms (humans, medaka, zebrafish, and common carp from Ensembl 78) were incorporated into MAKER2 to generate homologous gene structures (Cantarel et al., 2008). All predicted gene structures were integrated into the consensus gene models using MAKER2 (Cantarel et al., 2008). The gene models with high N contents (larger than 20%), without start or stop codon, or with codon number less than 50 were excluded in the prediction. CEGMA (Core Eukaryotic Gene Mapping Approach) was used to evaluate the gene region coverage (Parra, Bradnam, & Korf, 2007).

To determine the functional annotation of the gene models, a BLASTP search with an E‐value ≤1e^−5^ was performed against protein databases, including NR (nonredundant protein sequences in NCBI), Swiss‐Prot, KEGG (Kyoto Encyclopedia of Genes and Genomes database; Kanehisa, Sato, Kawashima, Furumichi, & Tanabe, 2015), RefSeq (Pruitt et al., 2014), and Trembl (Consortium, 2015). The resulting NR BLASTP hits were processed by BLAST2GO (Conesa et al., 2005) to retrieve associated Gene Ontology (GO) terms describing biological processes, molecular functions, and cellular components (E‐value ≤1e^−5^).

### 2.6 Mapping, variant calling, and demographics

To identify SNPs, we first used the BWA program to map the Illumina clean reads to the assembled contigs of corresponding species with default parameters. The “mpileup” module (with parameters: ‐q 1 –C 50 –g –t DP, SP –m 2) was then used to identify single nucleotide polymorphisms (SNPs) and short INDELS (Li & Durbin, 2010; <10 bp). VCFTOOLS was used to filter raw variants according to the sequencing depth of samples (parameters: vcfutils.pl varFilter –Q 20 –d 5 –D 250 –w 5 –W 10; Li et al., 2009). Single nucleotide polymorphisms between two sets of bighead carp and silver carp diploid genomes were identified. Nonoverlapping 50 kb windows were chosen, and the heterozygosity density was calculated (sequences <50 kb were excluded). Demographic histories of the bighead and silver carps were reconstructed using the Pairwise Sequentially Markovian Coalescent (PSMC) model (Li & Durbin, 2011) with the mutation rate of 0.2 × 10^–8^ per generation.

### 2.7 Genome evolution

#### 2.7.1 Identification of gene families

Protein sequences of 10 species of fishes (spotted gar, cavefish, zebrafish, common carp, Atlantic cod, takifugu, tetraodon, tongue sole, platyfish, and medaka) were downloaded from Ensembl (release version 78) and NCBI. Only the longest transcript was selected for each gene locus with alternative splicing variants. The genes that encode a protein with less than 50 amino acids were removed. The protein sequences from different species were compared using BLASTP with an E‐value of 1e^−5^, and low‐quality hits (identity <30% and coverage <30%) were removed. Orthologous groups were constructed by ORTHOMCL v2.0.9 (Chen, Mackey, Stoeckert, & Roos, 2006a) using default settings based on the filtered BLASTP results.

#### 2.7.2 Phylogenetic tree construction

Single‐copy gene families retrieved from the ORTHOMCL result were used for phylogenetic tree construction. The families containing any sequences shorter than 200 amino acids were removed. The protein sequences from each family were aligned using MUSCLE v3.8.31 (Edgar, 2004), and the corresponding CDS alignments were back‐translated from the corresponding protein alignments. The conserved CDS alignments were extracted by Gblocks (Talavera & Castresana, 2007). The resulting CDS alignments of each family were used for further phylogenomic analysis. For phylogenetic tree construction, CDS alignments of every single family were concatenated to generate a matrix of supergenes and fourfold synonymous (degenerative) third‐codon transversion (4DTV) sites extracted from the supergenes were used for the phylogenetic tree construction. MrBayes 3.22 was used to generate a Bayesian tree with the GTR + I + Γ model using 4DTV sites (Huelsenbeck & Ronquist, 2001). The MCMC process was run 5,000,000 generations, and trees were sampled every 100 generations with first 10,000 samples dropped.

#### 2.7.3 Divergence time estimation

The concatenated supergenes were separated into three partitions corresponding to the 1st, 2nd, and 3rd codon site in the CDS. Divergence times were estimated under a relaxed clock model using the MCMCTREE program in the PAML4.7 package (Yang, 2007). Independent rates model (clock = 2) and JC69 model in MCMCTREE program were used in the calculation (Yang, 2007). The MCMC process was run for 6,000,000 iterations after a burn‐in of 2,000,000 iterations. We ran the program twice for each dataset to confirm that the results were similar between runs. The following constraints were used for time calibrations: medaka—stickleback, takifugu, tetraodon (min 96.9 Mya; max 150.9 Mya); zebrafish—medaka, stickleback, takifugu, tetraodon (min 149.85 Mya; max 165.2 Mya); zebrafish, medaka, stickleback, takifugu, tetraodon—toad, bird, mammal (min 416 Mya; max 421.75 Mya), with 416 Mya assigned as the max age for ray‐finned fish (Hedges, Dudley, & Kumar, 2006).

#### 2.7.4 Positive selection analysis

The branch‐site model of CODEML in PAML4.7 (Yang, 2007) was applied to test potentially positively selected genes (PSGs), with the settings of bighead and silver carps as the foreground branch and the others as background branches. The likelihood ratio test was performed using the *χ* ^2^ statistic to calculate the *p*‐value and corrected the *p*‐values for multiple testing by the false discovery rate test with the Bonferroni correction to identify PSGs that met the requirements of corrected *p*‐value < .05. Significantly over‐represented GO terms among these PSGs were identified using topGO (Alexa & Rahenfuhrer, 2010).

#### 2.7.5 Branch‐specific dN/dS values

The branch‐specific selection was estimated based on the CDS alignments of each single‐copy gene family with reliable codons using the free‐ratios model and an F3x4 codon frequency model implemented by the CODEML program in PAML4.7 (Yang, 2007). The dN/dS values for each terminal branch were then fetched and plotted.

#### 2.7.6 Identification of expanded and contracted gene families

Expansion and contraction of gene families were characterized by comparing the cluster size of the ancestor to that of each of the current species using CAFÉ 3.1 (De Bie, Cristianini, Demuth, & Hahn, 2006).

### 2.8 Whole‐genome alignments

We evaluated the genomic similarity of both carps based upon whole‐genome alignments, which was conducted using the lastz program (Harris, 2007). The lastz outputs in the axt format were chained by the axtChain program. The chained alignments were processed into nets with chainNet and netSyntenic (Harris, 2007). Best‐chain alignments in axt format were extracted by the netToAxt program (Harris, 2007). These whole‐genome alignments were prepared for downstream analysis. We mapped contig sequences of bighead carp and silver carp to zebrafish chromosomes and then linked these mapped contigs to pseudo‐chromosomes according to the shared synteny to each zebrafish chromosome.

### 2.9 Effects of heterozygosity in hybrids

Single nucleotide polymorphisms in the genomes of hybrids were detected using the bighead carp genome as a reference with the BWA program (Li & Durbin, 2010). Functional prediction of the resultant nonsynonymous SNPs was conducted using snpEff (Cingolani et al., 2012), whereas the functional effects of these missense variants in hybrids were further evaluated by SIFT (Kumar, Henikoff, & Ng, 2009) and PolyPhen2 (Adzhubei et al., 2010). Mutations with SIFT score <0.05 are considered as potentially deleterious. PolyPhen‐2 uses a cutoff of <5% FPR for probably damaging mutations. PolyPhen‐2 prediction models were tested and trained using two pairs of datasets, HumanDiv and HumanVar.

### 2.10 Cross experiments

We conducted a cross experiment to evaluate gametic compatibility and hybrid viability, which allows us to explore the role of hybridization in the Asian carp invasion. Asian carp are invasive species, and live fish are prohibited to transport or possess in the United States. Thus, we conducted the experiment in their native country, that is, China. Four crosses were conducted in the Hanjiang National Four Major Chinese Carps Seed Farm, Jiangsu, China, on May 2012, including pure bighead and silver carps, and reciprocal hybrids, using three replicates for each cross. The number of fertilized versus unfertilized eggs, hatched versus unhatched embryos, and normal versus abnormal larvae was estimated following the standard protocols (Yi, Liang, Yu, Lin, & He, 1988). The eggs were photographed at different embryonic developmental stages. Approximately 30 fertilized eggs were sampled during stage 1 to stage 10 of embryonic development, and another 30 during stage 11 to stage 30. The significance tests were conducted using SPSS17.0.

## 3 RESULTS

### 3.1 Genome assembly and annotation

The Illumina HiSeq 2000 system generated 75 and 80 Gb short reads, whereas the PacBio RS II system produced 8.6 and 8.5 Gb long reads for bighead and silver carps, respectively. The reads were assembled into 661,239 scaffolds in bighead carp with an N50 length of 83 kb and 419,157 scaffolds in silver carp with an N50 of 315 kb (Table S2). The genome size was approximately 1.08 Gb in bighead carp and 1.15 Gb in silver carp (Table S2). The repeated sequences were found to account for 43.5% of the genome in bighead carp and 35.2% in silver carp, with DNA transposons comprising more than 50% of the repeats in both species (Tables S3‐S4, Figure S1). Gene prediction analysis resulted in 26,516 protein‐coding genes in bighead carp and 26,880 genes in silver carp (Table S5). Approximately 97% of predicted genes had homologous proteins in public repositories such as Swiss‐Prot and NCBI NR. More than 70% of the translated proteins were functionally assigned to KEGG pathways and Gene Ontology (GO) categories (Table S5). The gene set assessment with CEGMA identified 94.4% and 96% ultra‐conserved core eukaryotic genes with partial sequences respectively in the genomes of bighead and silver carps, suggesting our assemblies captured the majority of protein‐coding sequences in both genomes (Table S6‐S7).

### 3.2 Genomic heterozygosity and population history

**Figure 1:**
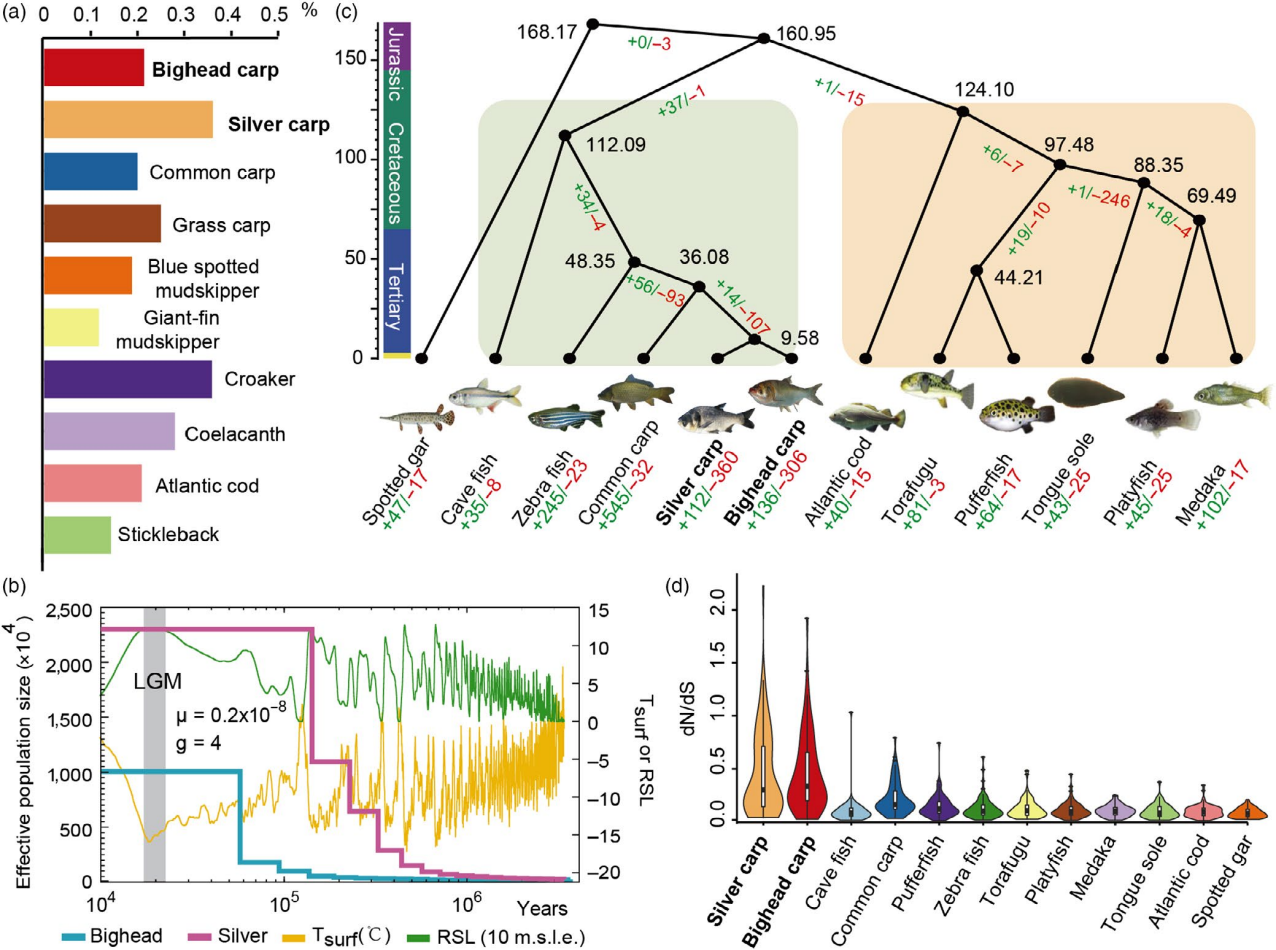
Genetic diversity, divergence, demographic history, and selection test of bighead and silver carp. (a) The heterozygosity rates of 10 fish species. (b) Inferred population history of bighead carp (bighead) and silver carp (silver) by the PSMC. The last glacial maximum (LGM) is highlighted in gray. Tsurf, atmospheric surface air temperature; RSL, relative sea level. (c) Phylogenetic positions of bighead and silver carps relative to other fishes, with the number of species/clade‐specific, expanded gene families (green), the number of species/clade‐specific contracted gene families (red), and the divergence time (Mya, black). (d) The dN/dS ratios of 950 1:1 orthologous genes in 12 ray‐finned fishes

We detected approximately 1.92 and 2.96 million single nucleotide polymorphisms (SNPs) in bighead and silver carps, respectively. The genomic heterozygosity was estimated to be 0.0021 in bighead carp and 0.0036 in silver carp (Table S8). The heterozygosity level was considered moderate (bighead carp) and high (silver carp) when compared to other species of fish (Figure 1a). The population history inferred from the draft genomes showed the effective population size increased approximately one million years ago (Mya) and had become relatively stable since 55,000 (bighead carp) and 150,000 (silver carp) YA (Figure 1b). In addition, the population size appeared to be twice as large for silver carp compared to bighead carp during the past 55,000 years.

The genomic analysis of 12 ray‐finned fishes identified 950 single‐copy orthologous genes. The alignment of these single‐copy genes resulted in a supermatrix of 660,222 nucleotide positions, which was used for phylogenetic tree reconstruction and molecular dating. Figure 1c shows the phylogenetic positions of bighead and silver carps relative to other fishes. Approximately 136 and 112 gene families were found to have experienced expansion in bighead carp and silver carp, respectively, whereas 306 and 360 gene families underwent contraction since their divergence around 9.6 Mya. Selection tests on these single‐copy genes demonstrated the dN/dS ratios are significantly higher (Wilcoxon test, *p* < .000001) in bighead and silver carps compared to other ray‐finned fishes (Figure 1d, Table S9).

### 3.3 Genes under strong positive selection

Among the 950 single‐copy genes, 252 significant positive selection genes were identified in bighead carp, 254 in silver carp, and 43 common genes in both carps (Table 1). Functional analysis showed these consensus genes are involved in growth and development (e.g., methionine synthase and malcavernin), environmental adaptation (*Metrnl*), and sperm mobility (*tektin‐2*). In particular, genes such as *14‐alpha‐demethylase*, *squalene synthase*, and *mevalonate kinase* that play an important role in the terpenoid backbone biosynthesis of the mevalonate pathway, an important pathway associated with food habit transition in grass carp, were found in the genomes of both bighead and silver carps.

**Table 1:** Positive selection genes found in the genomes of bighead and silver carps and their corresponding functions

Gene	Description	Putative function
*METRNL*	Meteorin‐like protein precursor	A role in metabolic adaptations to cold temperatures
*ZNF385A*	Zinc finger protein 385A isoform X2	May play a role in adipogenesis through binding to the 3′‐UTR of CEBPA mRNA and regulation of its translation
*TEKT2*	Tektin‐2	Plays a key role in the assembly or attachment of the inner dynein arm to microtubules in sperm flagella and tracheal cilia
*IFT52*	Intraflagellar transport protein 52 homolog	Essential for spermiogenesis
*RAB10*	Ras‐related protein Rab‐10	May play a role in endoplasmic reticulum dynamics and morphology controlling tubulation along microtubules and tubule fusion
*TMBIM1*	Protein lifeguard 3	May play a protective role in vascular remodeling
*NLE1*	Notchless protein homolog 1	Required during embryogenesis for inner mass cell survival
*SLC25A14*	Brain mitochondrial carrier protein 1‐like isoform X1	Participates in the mitochondrial proton leak measured in brain mitochondria
*CCM2*	Malcavernin	Maintain normal blood vessel structure
*RHAG*	Rhesus blood group‐associated glycoprotein A‐like protein	An ammonia transporter protein
*PHF10*	PHD finger protein 10	Required for the proliferation of neural progenitors
*CHST10*	Carbohydrate sulfotransferase 10 isoform X5	Transfer sulfate to carbohydrate groups in glycoproteins and glycolipids
*KDSR*	3‐Ketodihydrosphingosine reductase‐like	Acting on the CH‐OH group of donor with NAD+ or NADP+ as acceptor
*CDC5L*	cell division cycle 5‐like protein	DNA‐binding protein involved in cell cycle control
*DPH6*	Diphthine–ammonia ligase isoform X1	Amidase that catalyzes the last step of diphthamide biosynthesis using ammonium and ATP
*DNAJC17*	DnaJ homolog subfamily C member 17	May negatively affect PAX8‐induced thyroglobulin/TG transcription
*GPATCH2*	G patch domain‐containing protein 2 isoform X1	May play a role in mRNA splicing
*GATAD1*	GATA zinc finger domain‐containing protein 1	Component of some chromatin complex recruited to chromatin sites methylated “Lys‐4” of histone H3 (H3K4me)
*PGAP1*	GPI inositol‐deacylase	Involved in inositol deacylation of GPI‐anchored proteins
*Mcm2*	DNA replication licensing factor MCM2	Required for DNA replication and cell proliferation
*THAP4*	THAP domain‐containing protein 4	DNA binding and metal ion binding
*MTR*	Methionine synthase	Regenerate Met in the S‐Adenosyl methionine cycle
*FDXR*	NADPH:adrenodoxin oxidoreductase, mitochondrial	Serves as the first electron transfer protein in all the mitochondrial P450 systems
*PTCD3*	Pentatricopeptide repeat domain‐containing protein 3, mitochondrial precursor	Plays a role in mitochondrial translation
*FKBP8*	peptidylprolyl cis‐trans isomerase FKBP8	Plays a role in the regulation of apoptosis
*Ppwd1*	peptidylprolyl isomerase domain and WD repeat‐containing protein 1	May be involved in pre‐mRNA splicing
*PPAT*	Phosphoribosyl pyrophosphate amidotransferase	Involved in *de novo* purine synthesis
*POU6F2*	POU domain, class 6, transcription factor 2	Involved in early steps in the differentiation of amacrine and ganglion cells
*PQLC1*	PQ‐loop repeat‐containing protein 1	Membrane‐bound proteins
*mkrn1*	Probable E3 ubiquitin‐protein ligase makorin‐1	Catalyzing the covalent attachment of ubiquitin moieties onto substrate proteins
*ITFG3*	Protein ITFG3	Membrane proteins
*SUPT5H*	Transcription elongation factor SPT5	Component of the DRB sensitivity‐inducing factor complex (DSIF complex)
*PAF1*	RNA polymerase II‐associated factor 1 homolog	Regulation of development and maintenance of embryonic stem cell pluripotency
*ULK3*	serine/threonine‐protein kinase ULK3	Able to induce autophagy
*smpd5*	sphingomyelin phosphodiesterase 5	Catalyzes the hydrolysis of membrane sphingomyelin to form phosphorylcholine and ceramide
*VAT1*	Synaptic vesicle membrane protein VAT‐1 homolog‐like	Plays a part in calcium‐regulated keratinocyte activation in epidermal repair mechanisms
*TBC1D30*	TBC1 domain family member 30 isoform X4	GTPase activator activity and Rab GTPase binding
*TSPAN7*	Tetraspanin‐7	May be involved in cell proliferation and cell motility
*TM4SF18*	Transmembrane 4 L six family member 18 isoform X1	Multi‐pass membrane protein
*TMED6*	Transmembrane emp24 domain‐containing protein 6‐like	Involved in protein trafficking and secretion
*VPS41*	Vacuolar protein sorting‐associated protein 41 homolog	Plays a role in vesicle‐mediated protein trafficking to lysosomal compartments
*LMAN2*	Vesicular integral‐membrane protein VIP36 isoform X1	Plays a role as an intracellular lectin in the early secretory pathway
*pus10*	Pseudouridylate synthase	Synthesis of pseudouridine from uracil‐54 and uracil‐55

### 3.4 Species‐specific genes and gene families

**Figure 2:**
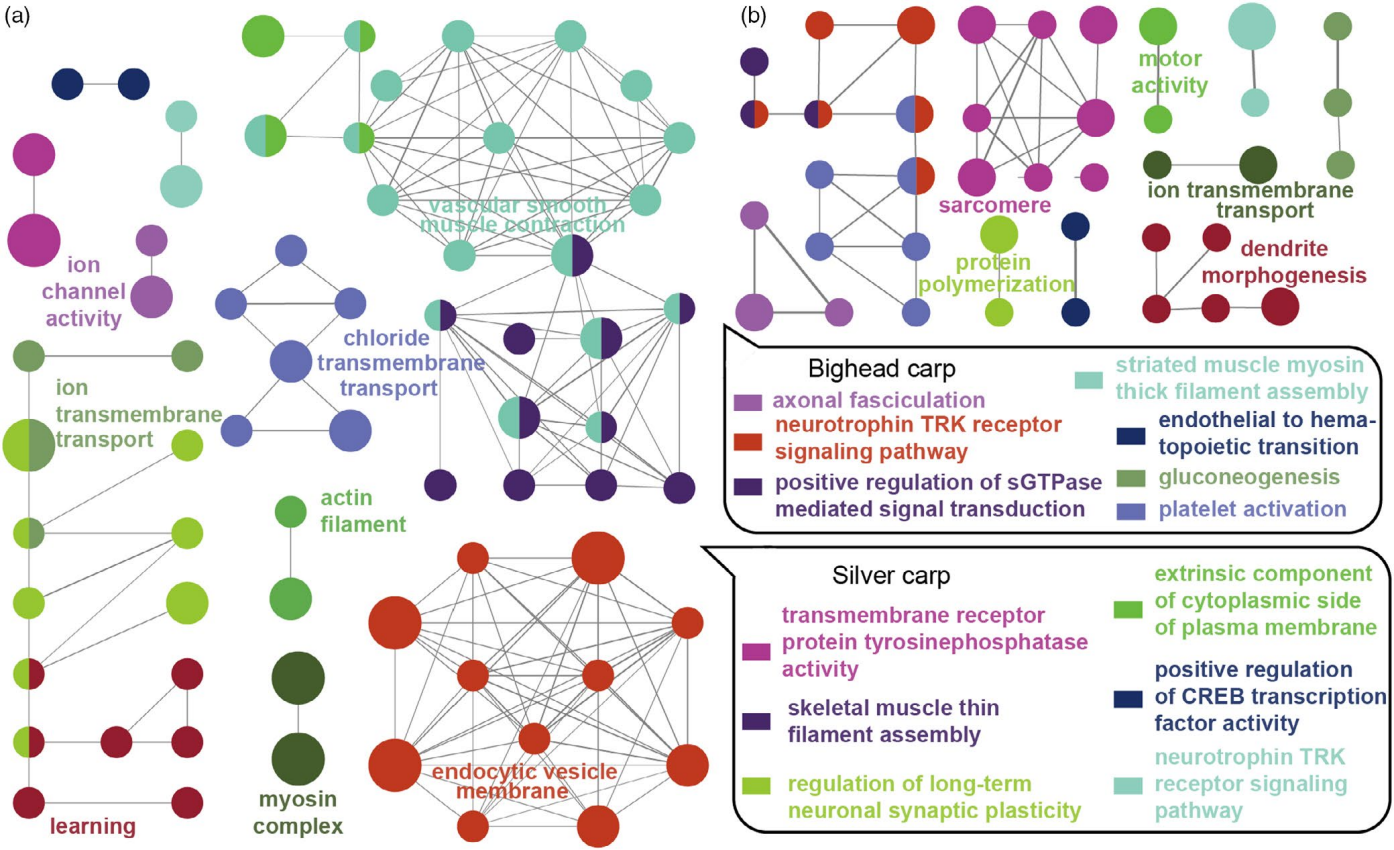
Functional Gene Ontology groups of species‐specific genes in silver carp (a) and bighead carp (b) analyzed with the ClueGO plugin of Cytoscape. For each group, only the GO terms with corrected *p*‐values ≤.05 are shown, and the major significant GO term is selected as the representation of that group. The significance of the GO term is reflected by the size of the nodes

The comparison of species‐specific gene families identified 21 gene families undergoing contraction in bighead carp, but expansion in silver carp (Table 2). These gene families are mostly associated with the action potential calcium channel and cardiac muscle function (Table 2). We also identified 172 species‐specific genes in bighead carp and 225 in silver carp. As shown in Figure 2, the bighead carp‐specific genes are enriched in molecular functions, such as striated muscle myosin thick filament assembly, axonal fasciculation, and gluconeogenesis (Figure 2b, Table S10, Figure S2‐S3), whereas silver carp‐specific genes were enriched in molecular functions, such as vascular smooth muscle contraction and endocytic vesicle membrane (Figure 2a, Table S11, Figure S4‐S5).

**Table 2:** Gene families under expansion in silver carp yet under contraction in bighead carp

GO terms	Silver expansion	Bighead contraction
Calcium ion binding	0.011648	1.90E−26
Calcium ion transmembrane transport	1.50E−11	0.002893
Calcium‐mediated signaling using intracellular calcium source	0.001641	0.005166
Cardiac muscle hypertrophy	0.033024	0.0323
Cellular calcium ion homeostasis	1.69E−09	0.003621
Cellular response to caffeine	8.33E−07	0.003621
Detection of calcium ion	0.00164	0.007427
Fast‐twitch skeletal muscle fiber contraction	0.010923	0.006061
Inositol 1,4,5‐trisphosphate‐sensitive calcium‐release channel activity	1.86E−06	0.000514
Larval locomotory behavior	3.09E−02	0.000386
Positive regulation of heart rate	1.64E−03	0.014897
Positive regulation of ryanodine‐sensitive calcium‐release channel activity	1.32E−04	0.007427
Protein kinase A catalytic subunit binding	1.44E−03	0.003483
Protein kinase A regulatory subunit binding	1.44E−03	0.003483
Protein self‐association	1.32E−04	0.018301
Regulation of cardiac muscle contraction by regulation of the release of sequestered calcium ion	1.32E−04	0.018301
Response to redox state	4.57E−04	0.002655
Ryanodine‐sensitive calcium‐release channel activity	1.06E−20	6.33E−13
Sarcoplasmic reticulum membrane	1.32E−04	0.007427
Smooth endoplasmic reticulum	3.96E−03	0.011005
Ventricular cardiac muscle cell action potential	1.30E−03	0.001653

### 3.5 Genome compatibility and hybrid viability

**Figure 3:**
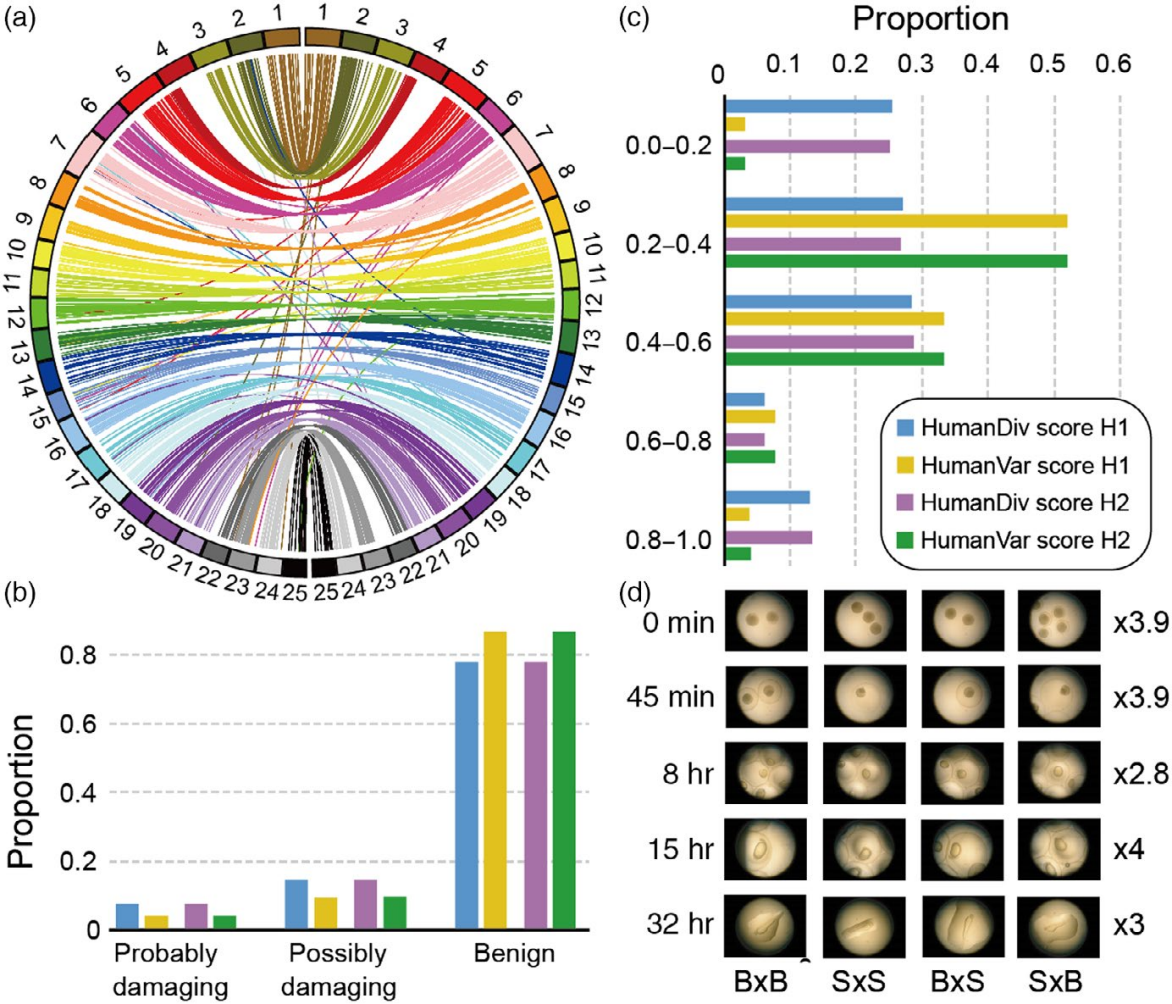
Genome compatibility between bighead and silver carps and functional prediction of nonsynonymous mutations in hybrids. (a) Circos plot of syntenic blocks in pseudo‐chromosomes between the genomes of bighead and silver carps with zebrafish genome as a reference and each pseudo‐chromosome color‐coded. (b) Functional effects of nonsynonymous SNPs predicted in F_1_ hybrids by PolyPhen‐2 under HumanDiv and HumanVar models. (c) Functional effects of nonsynonymous SNPs in F_1_ hybrids predicted by SIFT under HumanDiv and HumanVar models. (d) The embryonic development of pure (B ♀ × B ♂, S ♀ × S ♂) and hybrid (B ♀ × S ♂, S ♀ × B ♂) bigheaded carps. Images were taken from 0 to 32 hr after fertilization using a microscope with the magnification denoted

The pairwise comparison of 627,796 syntenic blocks showed a 96.31% genomic similarity between bighead carp and silver carp. The genomic sequencing of two F_1_ hybrids generated 80 Gb of sequence reads (Table S1). Mapping these reads to the assembled genomes of bighead and silver carps resulted in 6.58 and 7.92 million SNPs, respectively, in the two hybrids (Table S8). Functional prediction analysis showed the majority of nonsynonymous SNPs in F_1_ hybrids were benign (Figure 3b,c, Table S12‐S13). Our cross experiment showed a high fertilization rate between bighead and silver carps and high embryonic viability of F_1_ hybrids (Figure 3d). The fertilization rate was generally higher than 90%, and the hatch rate exceeded 96% in all crosses, with no significant difference among different crosses (Table S14). We also found low rates of larval deformation (<3%) in both pure and hybrid groups (Table S14), indicating the hybrids may have comparable viability in embryonic development to their parental species under experimental conditions.

## 4 DISCUSSION

### 4.1 Genome sequencing and assembly

The genomes of invasive bighead and silver carps we assembled are considered standard drafts based upon the community‐defined categories (Chain et al., 2009), but high quality according to the CEGMA assessment (Parra et al., 2007). The assembled genomes of both carps were predicted to possess a comparable number of protein‐coding genes as in zebrafish and grass carp (Howe et al., 2013; Wang et al., 2015), with around 97% of predicted genes having homologs in public protein repositories. The proportion of repeated sequences in the genomes of bigheaded carps appears to be different, but within the range reported in the genomes of other cyprinids, for example, 31.23% in common carp (Xu et al., 2014) and 57.09% in zebrafish (Howe et al., 2013).

Although both draft genomes are of good quality, further improvements of the assemblies are needed when comparing the genome assembly statistics between bigheaded carps and other species of fish such as zebrafish (Howe et al., 2013), fugu (Christoffels et al., 2004), and grass carp (Wang et al., 2015). More Illumina short reads from larger insert size libraries could improve the current bighead carp genome assembly, whereas more reads from small insert size libraries could enhance the draft genome of silver carp. This is because the contig N50 is higher in bighead carp than in silver carp, whereas the scaffold N50 is higher in silver carp. Alternatively, if the long‐read sequencing technologies, such as Pacific Biosciences (Eid et al., 2009) or Oxford Nanopore (Jain, Olsen, Paten, & Akeson, 2016), are used, more read coverages can help fill large gaps and correct misassemblies in the draft genomes. Additionally, integration of the draft genomes with high‐quality genetic maps of bigheaded carps (Fu, Liu, Yu, & Tong, 2016; Guo et al., 2013) would allow the genomes to be assembled at the chromosomal level, thereby improving the genome assemblies.

### 4.2 Genomic features and relevance to invasion establishment

We revealed genomics features, such as moderate‐to‐high genomic heterozygosity and elevated dN/dS ratios of single‐copy orthologous genes in bigheaded carps that could be resulted from rapid evolution following introduction, multiple introductions, and preintroduction adaptation within native ranges. Rapid evolution following introduction has been recognized as a common phenomenon in a variety of invasive organisms (Bock et al., 2015; Chown et al., 2015). The relatively high degree of genomic heterozygosity observed in invasive bigheaded carps could originate from mutation‐associated adaptation in novel environments; however, this scenario is less probable. The beneficial mutations that may occur in introduced environments often require time for occurrence and fixation (Bock et al., 2015). In fact, only a limited number of reproductive generations (some 10 generations) have been produced since the late 1970s (silver carp) or the early 1980s (bighead carp; Kolar et al., 2007). Further genomic investigation of population samples from both native and invasive ranges and across many different years may allow us to evaluate whether rapid evolution following introduction plays a role in the invasions of bigheaded carps in the MRB.

Multiple introductions can increase genetic diversity and variation of founding populations and have been identified as an important mechanism in many invasive species (Dlugosch & Parker, 2008; Facon et al., 2008; Kolbe et al., 2004). Bighead and silver carps were introduced from at least two sources, Taiwan and Yugoslavia (Kolar et al., 2007). It is possible that the relatively high genomic heterozygosity in invasive bighead and silver carps is attributed to multiple introductions of these species from different regions. However, this scenario requires further population genetic assessment of bigheaded carps in the MRB.

Many invasive species possess life history characteristics that contribute to their invasion success, such as maximum fecundity and propagule pressure (Baker & Stebbins, 1965; Kolar & Lodge, 2001). For bigheaded carps in the MRB, their invasion success is likely attributed to their rapid growth, high fecundity, and filter‐feeding behavior. These characteristics exist in native bigheaded carps (Li et al. 1990) and have evolved over the past millions of years. Therefore, it is likely the relative high genomic heterozygosity and high dN/dS ratios in invasive bigheaded carps are intrinsic features present from preintroduction adaptation within native ranges.

This study identified positively selected genes that are potentially associated with bigheaded carp life history traits and environmental adaptation, which supports the preintroduction adaptation hypothesis in invasions. Bighead and silver carps are traditionally characterized as opportunistic omnivores and can shift between zooplankton, phytoplankton, and detritus depending on the availability of food resources in the environment. This adaptive feeding strategy allows them to exploit multiple resources and novel environments (Anderson, Chapman, & Hayer, 2016; Cremer & Smitherman, 1980). We identified several positive selection genes in the mevalonate pathway in terpenoid backbone biosynthesis that have been associated with the transition between carnivorous and herbivorous feeding in grass carp (Wang et al., 2015). Furthermore, an important food resource of bighead and silver carps is cyanobacteria, especially *Microcystis* spp., which produces a class of toxins called microcystins (Zhang et al., 2006). Microcystins can cause cell death and DNA damage due to its inhibition of catalytic subunits of protein phosphatase and induction of ROS (reactive oxygen species; Cox & Goessling, 2015). We identified multiple positive selection genes in bighead and silver carps that are associated with microcystin detoxification (Chen, Xie, Zhang, Ke, & Yang, 2006b). We also found that the gene *tektin‐2* was also under positive selection. *Tektin‐2* is associated with sperm mobility (Bhilawadikar et al., 2013; Shimasaki et al., 2010), and its function may be related to improving reproductive success by increasing sperm mobility, thereby increasing fertilization rate and fecundity. High fecundity is an influential life history characteristic for determining establishment success (Baker & Stebbins, 1965; Kolar & Lodge, 2001).

### 4.3 Hybridization and relevance to invasion expansion

Interspecific hybridization can act as an evolutionary stimulus to promote invasions (Baker & Stebbins, 1965; Ellstrand & Schierenbeck, 2000; Mesgaran et al., 2016). In the case of bighead and silver carps, field surveys suggested both carps had already established reproductive populations during the late 1980s and the early 1990s in several states including Arkansas, Illinois, and Missouri (Kolar et al., 2007). Hybrids were not discovered until the late 1990s, suggesting hybrids between bigheaded carps were not prevalent in the initial introductions (Chapman, unpublished). Hybridization is most likely a fashion to facilitate their invasion success. Hybridization in these founder populations may have alleviated negative effects of low genetic diversity commonly observed in hatchery populations and low propagule pressure. The diversity of recombinant genotypes produced through hybridization may have increased the speed of evolution and added the genetic resiliency needed for these species to adapt and establish high‐density populations throughout the MRB. This is further supported by the high proportion of later generation hybrids in the system, indicating that hybridization has been occurring for a long time and a larger percentage of early‐generation hybrids likely once persisted in the population. This postintroduction introgression mechanism, coupled with preadaptation, has likely contributed to the invasion success of bigheaded carps in the MRB.

Natural hybrids of bighead and silver carps have rarely been reported in their native country, that is, China (Kolar et al., 2007), despite being highly pervasive in the MRB. Our in‐laboratory cross experiments revealed high fertilization rates in all crosses and high embryonic viability in F_1_ hybrids between native bigheaded carps. This strongly suggests prezygotic reproductive isolation (ecological or behavioral) may occur in native populations and is reinforced by evidence of strong reproductive potential of hybrids in the MRB (Lamer et al., 2019). Such temporal or spatial reproductive isolation that likely operates in native populations was likely lost in the MRB, resulting in an extensive hybridization between bighead and silver carps in the absence of environmental cues present in their native range. However, it should be noted that our cross experiments were conducted using bigheaded carps from their native region. Several studies have revealed significant genetic differences between native and invasive bigheaded carps (Farrington, Edwards, Bartron, & Lance, 2017; Li et al., 2011, 2010). Whether such genetic variation in invasive populations could lead to bias in hybrid fertilization rates and viability requires further investigation.

Genetic factors that support and restrict hybridization occur in bigheaded carps in the MRB. We showed a high genomic similarity between bighead and silver carps and the majority of nonsynonymous SNPs had no predicted functional effects on F_1_ hybrids. We determined that the fertilization rate and hatch success of hybrids were equal to that of parental species under experimental conditions; however, we were not able to determine the postzygotic effects throughout development. It has been observed in aquaculture that the offspring of F_1_ hybrids backcrossed with bighead carp exhibited apparent heterosis (The Yangtze River Fisheries Research Institute, 1975). Therefore, it is likely that the variability present within each individual F_1_ genome (equal contribution from each species) provides a source of variation and adaptability, but the rapid evolutionary potential occurs via additional introgression. Facilitated by highly extreme fecundity, each successive backcross provides an innumerable number of recombinant genotypes that can be molded by selection and isolation to produce highly adaptable and invasive species. Alternatively, F_1_ hybrids have been observed with deformed shapes or twisted gill rakers in the MRB, which may suggest possible hybrid inferiority (Kolar et al., 2007; Lamer et al., 2015), a likely explanation for the low percentage of F_1_ individuals found throughout the MRB (Lamer et al., 2015). Further investigations are needed to disentangle the genetic mechanisms underlying potential hybrid inferiority and hybrid vigor.

### 4.4 Applications and perspectives

The genome sequences of bighead and silver carps obtained in this study provide useful resources for applied research. Bigheaded carps are invasive species in the US and Canada and may have a severe impact on aquatic ecosystems and local fisheries. The US government has dedicated tremendous efforts to limiting the expansion of bigheaded carps and preventing their movement into the Great Lakes to protect a $7 billion fishing industry within the region (Cudmore, Mandrak, Dettmers, Chapman, & Kolar, 2012; Tsehaye, Catalano, Sass, Glover, & Roth, 2013). The predicted SNPs of bigheaded carps can be used for the development of more sensitive eDNA markers to monitor their invasion fronts, in particular, in the areas adjacent to the Great Lakes (Farrington et al., 2015; Stepien, Elz, & Snyder, 2019). We found silver carp‐specific genes enriched in biological processes that are likely linked to its jumping behavior. These species‐specific genes can be used to explore potential molecular or genetic control tools that may lead to mitigation of bigheaded carps in the MRB. From another perspective, bigheaded carps are among the most important aquaculture species in many Asian and some European countries (Li et al., 1990). The availability of the genomic resources in bigheaded carps makes it possible to develop molecular markers, in particular, those associated with quantitative traits for improved molecular selection and breeding of both species (Fu et al., 2016; Guo et al., 2013).

The invasive bighead and silver carps in the MRB that undergo extensive hybridization present an unprecedented model for the study of evolutionary processes and genetic consequences of a hybrid swarm. We know very little about the evolutionary dynamics of parental species and their hybrids and the fate of further genomic introgression between them. We showed previously in a transcriptomic study that F_1_ and backcrossed hybrids possessed pronounced variation in some Gene Ontology categories (Wang et al., 2016). Does this variation suggest the hybrids, even with the same genotypes, could have dissimilar fitness? If so, what would be the predicted population demographics of different parental and hybrid genotypes? It is essential for future studies to conduct a detailed genomic survey to build population models for this hybrid swarm and evaluate whether bigheaded carps in the MRB possess any heterotic genotypes, which will benefit management and control strategies of Asian carp in the MRB.

## 5 CONCLUSIONS

We described the draft genome sequences of two invasive Asian carp, bighead carp and silver carp, and presented their genomic features including heterozygosity and genes related to environmental adaptation and feeding habits. These intrinsic genomic features might have facilitated the early establishment of introduced bigheaded carps that escaped confinement and entered the Mississippi River Basin (MRB). In addition, this study identified hybrid bigheaded carps with high embryonic viability, which, along with the incidence of introgressive hybridization observed during the past two decades, suggests interspecific hybridization between bigheaded carps might have played an import role at the expansion stage of invasions in the MRB. Intrinsic genomic features and postintroduction hybridization might collectively contribute to the establishment and support continued invasions of bigheaded carps in the MRB, which thus reveals an alternative mechanism to provide additional insight into the genetic paradox of invasions.

## CONFLICT OF INTEREST

None declared.

## Data Availability

The NCBI BioProject accession numbers for genomic sequences are PRJNA305140 and PRJNA305141 (Wang et al., 2019).
