# Supplementary material for: Resolving the genetic paradox of invasions: Preadapted genomes and postintroduction hybridization of bigheaded carps in the Mississippi River Basin
Source: Evol Appl. 2019 Sep 12;13(2):263–77. doi: 10.1111/eva.12863 (PMC6976960; doi:10.1111/eva.12863)
Supplement: Supplementary file 1 [file EVA-13-263-s001.docx]

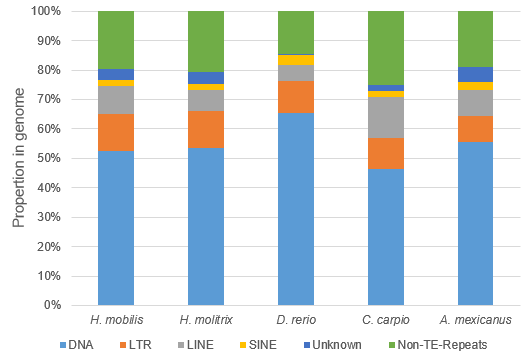


**Proportion in Genome**

**Figure S1.** The proportion of different types of repetitive DNA elements in the genomes of Cypriniform fishes: bighead carp (*H. mobiles*), silver carp (*H. molitrix*), zebrafish (*D. rerio*), common carp (*C. carpio*), blind cavefish (*A. mexicanus*).

**Figure S2.** Gene Ontology mapping of biological process for bighead carp specific genes

**Figure S3**. Gene Ontology mapping of molecular function for bighead carp specific genes

**Figure S4.** Gene Ontology mapping of biological process for silver carp specific genes

**Figure S5.** Gene Ontology mapping of molecular function for bighead carp specific gene
