# Supplementary material for: Resolving the genetic paradox of invasions: Preadapted genomes and postintroduction hybridization of bigheaded carps in the Mississippi River Basin
Source: Evol Appl. 2019 Sep 12;13(2):263–77. doi: 10.1111/eva.12863 (PMC6976960; doi:10.1111/eva.12863)
Supplement: Supplementary file 2 [file EVA-13-263-s002.docx]

**Table S1.** Genome sequencing platforms and libraries and the number of nucleotides generated for bighead carp, silver carp and their hybrids

| Platform | Library | Number of nucleotides (Gb) | | |
| --- | --- | --- | --- | --- |
|  |  | Bighead carp | Silver carp | Hybrids |
| Illumina HiSeq2000 | 170 bp pair-end | 36.0 | 33.0 |  |
|  | 450 bp pair-end | 24.0 | 22.0 |  |
|  | 2 Kb mate-pair | 9.0 | 12.0 |  |
|  | 5 Kb mate-pair | 6.0 | 13.0 |  |
|  | 170 bp pair-end |  |  | 65.0 |
|  | 2 Kb mate-pair |  |  | 15.4 |
| PacBio RS II | 20 Kb mate-pair | 8.6 | 8.5 |  |

**Table S2.** Genome assembly statistics in bighead carp and silver carp

| Assembly statistics | Bighead carp | Silver carp |
| --- | --- | --- |
| Number of scaffolds | 661,239 | 419,157 |
| Min scaffold (bp) | 200 | 200 |
| Max scaffold (bp) | 673,715 | 3,219,153 |
| Scaffold N50 (bp) | 83,188 | 314,534 |
| Contig N50 (bp) | 4,051 | 2,012 |
| Genome size (bp) | 1,080,337,275 | 1,146,467,876 |

**Table S3**. Repeat content in the genomes of Cypriniform fishes

| Species | Genome Size  (bp) | Count | Size (bp) | Repeat contents  (%, this study) | Repeat contents  (%, other studies) |
| --- | --- | --- | --- | --- | --- |
| Bighead carp (*H. nobilis*) | 1,080,337,275 | 4,250,697 | 470,139,019 | 43.52% |  |
| Silver carp (*H. molitrix*) | 1,146,467,876 | 4,063,216 | 403,240,721 | 35.17% |  |
| Zebrafish (*D. rerio*) | 1,412,464,843 | 4,300,021 | 806,421,164 | 57.09% | 52.2% ^1^  59.78% ^2^ |
| Common carp (*C. carpio*) | 1,713,686,240 | 4,419,779 | 645,537,851 | 37.67% | 37.0% ^2^ |
| Blind cave fish (*A. mexicanus*) | 1,191,242,572 | 3,272,666 | 419,963,054 | 35.25% | 30.08% ^3^ |

**Table S4.** Proportion of different subtypes of repeats in the genomes of Cypriniform fishes

| **Species** | **TE** | | | | | **Non-TE Repeats** |
| --- | --- | --- | --- | --- | --- | --- |
|  | **DNA** | **LTR** | **LINE** | **SINE** | **Unknown** |  |
| Bighead carp (*H. nobilis*) | 22.79% | 5.53% | 4.17% | 0.88% | 1.66% | 8.49% |
| Silver carp (*H. molitrix*) | 18.87% | 4.42% | 2.44% | 0.75% | 1.41% | 7.28% |
| Zebrafish (*D. rerio*) | 37.35% | 6.11% | 3.11% | 1.97% | 0.17% | 8.39% |
| Common carp (*C. carpio*) | 17.48% | 3.91% | 5.24% | 0.79% | 0.85% | 9.40% |
| Blind cave fish (*A. mexicanus*) | 19.54% | 3.18% | 3.06% | 0.99% | 1.83% | 6.66% |

Table S5. The number of gene models and proteins predicted and annotated

|  | **Bighead carp** | **Silver carp** |
| --- | --- | --- |
| Gene models | 26,516 | 26,880 |
| SwissProt | 23,907 | 24,337 |
| RefSeq | 25,630 | 25,964 |
| Trembl | 25,568 | 25,943 |
| NR | 25,647 | 25,990 |
| InterPro | 23,743 | 24,048 |
| KEGG | 18,695 | 19,026 |
| Gene Ontology | 19,164 | 19,450 |

Table S6. Statistics of the completeness of the bighead carp genome based on 248 CEGs*

|  | Prots | %Completeness | Total | Average | %Ortho |
| --- | --- | --- | --- | --- | --- |
| Complete | 207 | 83.47 | 301 | 1.45 | 33.82 |
| Group 1 | 49 | 74.24 | 64 | 1.31 | 26.53 |
| Group 2 | 47 | 83.93 | 70 | 1.49 | 34.04 |
| Group 3 | 54 | 88.52 | 81 | 1.5 | 35.19 |
| Group 4 | 57 | 87.69 | 86 | 1.51 | 38.6 |
|  |  |  |  |  |  |
| Partial | 234 | 94.35 | 411 | 1.76 | 47.01 |
| Group 1 | 61 | 92.42 | 97 | 1.59 | 44.26 |
| Group 2 | 52 | 92.86 | 88 | 1.69 | 42.31 |
| Group 3 | 59 | 96.72 | 113 | 1.92 | 47.46 |
| Group 4 | 62 | 95.38 | 113 | 1.82 | 53.23 |

*Prots: number of 248 ultra-conserved CEGs present in genome, %Completeness: percentage of 248 ultra-conserved CEGs present, Total: total number of CEGs present including putative orthologs, Average: average number of orthologs per CEG, %Ortho: percentage of detected CEGS that have more than 1 ortholog

Table S7. Statistics of the completeness of the silver carp genome based on 248 CEGs

|  | Prots | %Completeness | Total | Average | %Ortho |
| --- | --- | --- | --- | --- | --- |
| Complete | 209 | 84.27 | 266 | 1.27 | 22.97 |
| Group 1 | 55 | 83.33 | 66 | 1.2 | 16.36 |
| Group 2 | 44 | 78.57 | 54 | 1.23 | 22.73 |
| Group 3 | 49 | 80.33 | 64 | 1.31 | 24.49 |
| Group 4 | 61 | 93.85 | 82 | 1.34 | 27.87 |
|  |  |  |  |  |  |
| Partial | 238 | 95.97 | 346 | 1.45 | 34.87 |
| Group 1 | 65 | 98.48 | 88 | 1.35 | 29.23 |
| Group 2 | 54 | 96.43 | 79 | 1.46 | 40.74 |
| Group 3 | 56 | 91.8 | 85 | 1.52 | 35.71 |
| Group 4 | 63 | 96.92 | 94 | 1.49 | 34.92 |

*Prots: number of 248 ultra-conserved CEGs present in genome, %Completeness: percentage of 248 ultra-conserved CEGs present, Total: total number of CEGs present including putative orthologs, Average: average number of orthologs per CEG, %Ortho: percentage of detected CEGS that have more than 1 ortholog

**Table S8.** Genome variation of bighead carp, silver carp sampled from the Mississippi River Basin

| **Individual** | **Reference genome** | **Number of INDEL** | **Number of Heterozygous SNP** | **Number of Homologous SNP** | **Heterozygosity rate** |
| --- | --- | --- | --- | --- | --- |
| Bighead carp | Bighead carp | 95,4081 | 1,921,359 | 240,715 | 0.00214 |
| Silver carp | Bighead carp | 2,030,454 | 3,556,342 | 7,668,130 |  |
| Silver carp | Silver carp | 89,9679 | 2,963,037 | 289,154 | 0.00360 |
| F1 hybrid (H1) | Bighead carp | 1,531,496 | 7,915,962 | 736,936 |  |
| F1 hybrid (H2) | Silver carp | 1,254,027 | 6,575,823 | 863,230 |  |

**Table S9** Median dN/dS ratios in 12 ray-finned fishes

| **Common Name *(Latin Name)*** | **Median dN/dS** |
| --- | --- |
| Bighead carp *(H. nobilis)* | 0.34 |
| Silver carp *(H. molitrix)* | 0.30 |
| Mexican tetra  *(Astyanax mexicanus)* | 0.08 |
| Common carp *(Cyprinus carpio)* | 0.16 |
| Tongue sole *(Cynoglossus semilaevis)* | 0.08 |
| Zebrafish *(Danio rerio)* | 0.10 |
| Atlantic cod *(Gadus morhua)* | 0.09 |
| **Coelacanth** *(Latimeria chalumnae)* | 0.08 |
| Metaka *(Oryzias latipes)* | 0.10 |
| Pufferfish *(Tetraodon nigroviridis)* | 0.12 |
| Torafugu *(Takifugu rubripes)* | 0.10 |
| **Platyfish** (*Xiphophorus maculatus*) | 0.09 |

**Table S10**. Enriched Gene Ontology molecular function categories for unique genes in bighead carp

| GO | Class* | Annotated | Significant | Expected | P-value | Term |
| --- | --- | --- | --- | --- | --- | --- |
| GO:0001085 | MF | 47 | 12 | 6.18 | 1.20E-05 | RNA polymerase II transcription factor binding |
| GO:0003924 | MF | 256 | 58 | 33.68 | 1.90E-05 | GTPase activity |
| GO:0070742 | MF | 5 | 5 | 0.66 | 3.90E-05 | C2H2 zinc finger domain binding |
| GO:0005525 | MF | 481 | 94 | 63.27 | 4.20E-05 | GTP binding |
| GO:0019904 | MF | 309 | 82 | 40.65 | 5.20E-05 | protein domain specific binding |
| GO:0031432 | MF | 8 | 6 | 1.05 | 0.00011 | titin binding |
| GO:0003774 | MF | 223 | 47 | 29.33 | 0.0002 | motor activity |
| GO:0017124 | MF | 47 | 16 | 6.18 | 0.0002 | SH3 domain binding |
| GO:0030375 | MF | 6 | 5 | 0.79 | 0.00021 | thyroid hormone receptor coactivator activity |
| GO:0005234 | MF | 86 | 24 | 11.31 | 0.00021 | extracellular-glutamate-gated ion channel activity |
| GO:0000166 | MF | 2970 | 455 | 390.69 | 0.00032 | nucleotide binding |
| GO:0005200 | MF | 49 | 16 | 6.45 | 0.00035 | structural constituent of cytoskeleton |
| GO:0005515 | MF | 3828 | 578 | 503.56 | 0.00049 | protein binding |
| GO:0032403 | MF | 484 | 84 | 63.67 | 0.00059 | protein complex binding |
| GO:0004303 | MF | 14 | 7 | 1.84 | 0.00099 | estradiol 17-beta-dehydrogenase activity |
| GO:0017154 | MF | 18 | 8 | 2.37 | 0.00113 | semaphorin receptor activity |
| GO:0046966 | MF | 11 | 6 | 1.45 | 0.00131 | thyroid hormone receptor binding |
| GO:0001965 | MF | 8 | 5 | 1.05 | 0.00155 | G-protein alpha-subunit binding |
| GO:0035014 | MF | 12 | 6 | 1.58 | 0.00234 | phosphatidylinositol 3-kinase regulator activity |
| GO:0030898 | MF | 12 | 6 | 1.58 | 0.00234 | actin-dependent ATPase activity |
| GO:0044325 | MF | 50 | 14 | 6.58 | 0.00411 | ion channel binding |
| GO:0032550 | MF | 2242 | 339 | 294.93 | 0.00502 | purine ribonucleoside binding |
| GO:0005388 | MF | 22 | 8 | 2.89 | 0.00511 | calcium-transporting ATPase activity |
| GO:0043548 | MF | 22 | 8 | 2.89 | 0.00511 | phosphatidylinositol 3-kinase binding |
| GO:0003872 | MF | 10 | 5 | 1.32 | 0.00556 | 6-phosphofructokinase activity |
| GO:0005021 | MF | 18 | 7 | 2.37 | 0.00571 | vascular endothelial growth factor-activated receptor activity |
| GO:0035639 | MF | 2236 | 337 | 294.14 | 0.0069 | purine ribonucleoside triphosphate binding |
| GO:0004971 | MF | 23 | 8 | 3.03 | 0.00694 | alpha-amino-3-hydroxy-5-methyl-4-isoxazole propionate selective glutamate receptor activity |
| GO:0005230 | MF | 179 | 43 | 23.55 | 0.00718 | extracellular ligand-gated ion channel activity |
| GO:0017016 | MF | 134 | 28 | 17.63 | 0.00741 | Ras GTPase binding |
| GO:0016594 | MF | 7 | 4 | 0.92 | 0.00751 | glycine binding |
| GO:0022824 | MF | 7 | 4 | 0.92 | 0.00751 | transmitter-gated ion channel activity |
| GO:0004970 | MF | 88 | 25 | 11.58 | 0.00778 | ionotropic glutamate receptor activity |
| GO:0004714 | MF | 132 | 32 | 17.36 | 0.00778 | transmembrane receptor protein tyrosine kinase activity |
| GO:0017048 | MF | 52 | 14 | 6.84 | 0.00796 | Rho GTPase binding |
| GO:0015278 | MF | 23 | 7 | 3.03 | 0.00818 | calcium-release channel activity |
| GO:0046982 | MF | 240 | 45 | 31.57 | 0.00845 | protein heterodimerization activity |
| GO:0005080 | MF | 15 | 6 | 1.97 | 0.00892 | protein kinase C binding |
| GO:0004872 | MF | 1430 | 244 | 188.11 | 0.0091 | receptor activity |
| GO:0071688 | BP | 14 | 9 | 1.81 | 6.10E-07 | striated muscle myosin thick filament assembly |
| GO:0031444 | BP | 8 | 7 | 1.04 | 4.30E-06 | slow-twitch skeletal muscle fiber contraction |
| GO:0007411 | BP | 321 | 69 | 41.59 | 7.80E-06 | axon guidance |
| GO:0034220 | BP | 986 | 163 | 127.75 | 1.30E-05 | ion transmembrane transport |
| GO:0048011 | BP | 61 | 21 | 7.9 | 1.40E-05 | neurotrophin TRK receptor signaling pathway |
| GO:0030168 | BP | 86 | 24 | 11.14 | 4.20E-05 | platelet activation |
| GO:2000273 | BP | 16 | 9 | 2.07 | 4.90E-05 | positive regulation of receptor activity |
| GO:0042759 | BP | 10 | 7 | 1.3 | 5.10E-05 | long-chain fatty acid biosynthetic process |
| GO:0006112 | BP | 84 | 19 | 10.88 | 5.70E-05 | energy reserve metabolic process |
| GO:0035235 | BP | 91 | 26 | 11.79 | 6.00E-05 | ionotropic glutamate receptor signaling pathway |
| GO:0051258 | BP | 124 | 31 | 16.07 | 8.00E-05 | protein polymerization |
| GO:0048813 | BP | 79 | 27 | 10.24 | 8.00E-05 | dendrite morphogenesis |
| GO:0007413 | BP | 42 | 16 | 5.44 | 0.00018 | axonal fasciculation |
| GO:0002576 | BP | 15 | 8 | 1.94 | 0.00022 | platelet degranulation |
| GO:0030049 | BP | 12 | 7 | 1.55 | 0.00026 | muscle filament sliding |
| GO:0051057 | BP | 21 | 8 | 2.72 | 0.00028 | positive regulation of small GTPase mediated signal transduction |
| GO:0061333 | BP | 14 | 6 | 1.81 | 0.00028 | renal tubule morphogenesis |
| GO:0007156 | BP | 191 | 42 | 24.75 | 0.00036 | homophilic cell adhesion |
| GO:0090129 | BP | 16 | 8 | 2.07 | 0.00038 | positive regulation of synapse maturation |
| GO:0006184 | BP | 812 | 143 | 105.21 | 0.00043 | GTP catabolic process |
| GO:0051056 | BP | 468 | 84 | 60.64 | 0.00047 | regulation of small GTPase mediated signal transduction |
| GO:2000300 | BP | 7 | 5 | 0.91 | 0.00061 | regulation of synaptic vesicle exocytosis |
| GO:0034405 | BP | 10 | 6 | 1.3 | 0.00062 | response to fluid shear stress |
| GO:0002027 | BP | 54 | 18 | 7 | 0.00066 | regulation of heart rate |
| GO:0071526 | BP | 25 | 10 | 3.24 | 0.00067 | semaphorin-plexin signaling pathway |
| GO:0071300 | BP | 21 | 9 | 2.72 | 0.00069 | cellular response to retinoic acid |
| GO:0007268 | BP | 544 | 120 | 70.48 | 0.00085 | synaptic transmission |
| GO:0007017 | BP | 471 | 64 | 61.03 | 0.00088 | microtubule-based process |
| GO:0006094 | BP | 102 | 24 | 13.22 | 0.00091 | gluconeogenesis |
| GO:0043065 | BP | 144 | 32 | 18.66 | 0.00095 | positive regulation of apoptotic process |
| GO:0007186 | BP | 938 | 143 | 121.53 | 0.00096 | G-protein coupled receptor signaling pathway |
| GO:0001666 | BP | 113 | 29 | 14.64 | 0.001 | response to hypoxia |
| GO:0006096 | BP | 82 | 22 | 10.62 | 0.00107 | glycolytic process |
| GO:0051965 | BP | 31 | 11 | 4.02 | 0.00118 | positive regulation of synapse assembly |
| GO:0051491 | BP | 11 | 6 | 1.43 | 0.00121 | positive regulation of filopodium assembly |
| GO:0006703 | BP | 11 | 6 | 1.43 | 0.00121 | estrogen biosynthetic process |
| GO:0060391 | BP | 5 | 4 | 0.65 | 0.00126 | positive regulation of SMAD protein import into nucleus |
| GO:0007628 | BP | 8 | 5 | 1.04 | 0.00145 | adult walking behavior |
| GO:0032870 | BP | 324 | 60 | 41.98 | 0.00152 | cellular response to hormone stimulus |
| GO:0007214 | BP | 47 | 14 | 6.09 | 0.00189 | gamma-aminobutyric acid signaling pathway |
| GO:0042692 | BP | 329 | 62 | 42.63 | 0.00208 | muscle cell differentiation |
| GO:0017158 | BP | 25 | 10 | 3.24 | 0.00215 | regulation of calcium ion-dependent exocytosis |
| GO:0048532 | BP | 12 | 6 | 1.55 | 0.00216 | anatomical structure arrangement |
| GO:0060337 | BP | 12 | 4 | 1.55 | 0.00217 | type I interferon signaling pathway |
| GO:0097190 | BP | 214 | 34 | 27.73 | 0.00232 | apoptotic signaling pathway |
| GO:0030240 | BP | 20 | 8 | 2.59 | 0.00232 | skeletal muscle thin filament assembly |
| GO:0038084 | BP | 20 | 8 | 2.59 | 0.00232 | vascular endothelial growth factor signaling pathway |
| GO:0006013 | BP | 53 | 15 | 6.87 | 0.00232 | mannose metabolic process |
| GO:0038095 | BP | 39 | 12 | 5.05 | 0.0029 | Fc-epsilon receptor signaling pathway |
| GO:0055119 | BP | 15 | 7 | 1.94 | 0.0029 | relaxation of cardiac muscle |
| GO:0044699 | BP | 12671 | 1698 | 1641.74 | 0.00316 | single-organism process |
| GO:0046822 | BP | 71 | 14 | 9.2 | 0.00338 | regulation of nucleocytoplasmic transport |
| GO:0032413 | BP | 16 | 6 | 2.07 | 0.00339 | negative regulation of ion transmembrane transporter activity |
| GO:0051932 | BP | 20 | 6 | 2.59 | 0.00339 | synaptic transmission, GABAergic |
| GO:0045920 | BP | 6 | 4 | 0.78 | 0.00339 | negative regulation of exocytosis |
| GO:1902037 | BP | 6 | 4 | 0.78 | 0.00339 | negative regulation of hematopoietic stem cell differentiation |
| GO:1901018 | BP | 6 | 4 | 0.78 | 0.00339 | positive regulation of potassium ion transmembrane transporter activity |
| GO:0098508 | BP | 6 | 4 | 0.78 | 0.00339 | endothelial to hematopoietic transition |
| GO:0070059 | BP | 18 | 8 | 2.33 | 0.00356 | intrinsic apoptotic signaling pathway in response to endoplasmic reticulum stress |
| GO:0006904 | BP | 35 | 11 | 4.53 | 0.00357 | vesicle docking involved in exocytosis |
| GO:0006534 | BP | 17 | 7 | 2.2 | 0.0036 | cysteine metabolic process |
| GO:0030947 | BP | 26 | 9 | 3.37 | 0.00398 | regulation of vascular endothelial growth factor receptor signaling pathway |
| GO:0055010 | BP | 36 | 11 | 4.66 | 0.00455 | ventricular cardiac muscle tissue morphogenesis |
| GO:0031346 | BP | 115 | 29 | 14.9 | 0.00509 | positive regulation of cell projection organization |
| GO:0048041 | BP | 30 | 9 | 3.89 | 0.00519 | focal adhesion assembly |
| GO:1901998 | BP | 17 | 6 | 2.2 | 0.0052 | toxin transport |
| GO:0007274 | BP | 10 | 5 | 1.3 | 0.0052 | neuromuscular synaptic transmission |
| GO:0006171 | BP | 72 | 17 | 9.33 | 0.00526 | cAMP biosynthetic process |
| GO:0007612 | BP | 53 | 14 | 6.87 | 0.00527 | learning |
| GO:0006936 | BP | 163 | 48 | 21.12 | 0.00527 | muscle contraction |
| GO:0043524 | BP | 63 | 16 | 8.16 | 0.00552 | negative regulation of neuron apoptotic process |
| GO:0034162 | BP | 14 | 6 | 1.81 | 0.00557 | toll-like receptor 9 signaling pathway |
| GO:0002026 | BP | 23 | 8 | 2.98 | 0.00633 | regulation of the force of heart contraction |
| GO:0019233 | BP | 33 | 11 | 4.28 | 0.00689 | sensory perception of pain |
| GO:0022038 | BP | 7 | 4 | 0.91 | 0.0071 | corpus callosum development |
| GO:0003139 | BP | 7 | 4 | 0.91 | 0.0071 | secondary heart field specification |
| GO:0060080 | BP | 7 | 4 | 0.91 | 0.0071 | regulation of inhibitory postsynaptic membrane potential |
| GO:0051968 | BP | 7 | 4 | 0.91 | 0.0071 | positive regulation of synaptic transmission, glutamatergic |
| GO:0000187 | BP | 33 | 10 | 4.28 | 0.00715 | activation of MAPK activity |
| GO:0045471 | BP | 31 | 9 | 4.02 | 0.00738 | response to ethanol |
| GO:0071222 | BP | 29 | 8 | 3.76 | 0.00739 | cellular response to lipopolysaccharide |
| GO:0086001 | BP | 19 | 7 | 2.46 | 0.00782 | cardiac muscle cell action potential |
| GO:0042403 | BP | 10 | 4 | 1.3 | 0.00784 | thyroid hormone metabolic process |
| GO:0009068 | BP | 51 | 6 | 6.61 | 0.00788 | aspartate family amino acid catabolic process |
| GO:0007626 | BP | 123 | 32 | 15.94 | 0.00794 | locomotory behavior |
| GO:0050853 | BP | 15 | 6 | 1.94 | 0.00828 | B cell receptor signaling pathway |
| GO:0055005 | BP | 15 | 6 | 1.94 | 0.00828 | ventricular cardiac myofibril assembly |
| GO:0071320 | BP | 24 | 8 | 3.11 | 0.00843 | cellular response to cAMP |
| GO:0021762 | BP | 24 | 8 | 3.11 | 0.00843 | substantia nigra development |
| GO:0021559 | BP | 11 | 5 | 1.43 | 0.00853 | trigeminal nerve development |
| GO:0007512 | BP | 11 | 5 | 1.43 | 0.00853 | adult heart development |
| GO:0042921 | BP | 11 | 5 | 1.43 | 0.00853 | glucocorticoid receptor signaling pathway |
| GO:0006012 | BP | 45 | 12 | 5.83 | 0.0088 | galactose metabolic process |
| GO:0032879 | BP | 1027 | 174 | 133.06 | 0.00987 | regulation of localization |
| GO:0005886 | CC | 2375 | 438 | 307.46 | 7.70E-11 | plasma membrane |
| GO:0030425 | CC | 199 | 53 | 25.76 | 3.70E-06 | dendrite |
| GO:0016020 | CC | 6647 | 979 | 860.5 | 1.90E-05 | membrane |
| GO:0031093 | CC | 7 | 6 | 0.91 | 2.90E-05 | platelet alpha granule lumen |
| GO:0030016 | CC | 138 | 43 | 17.87 | 3.60E-05 | myofibril |
| GO:0030017 | CC | 116 | 31 | 15.02 | 5.20E-05 | sarcomere |
| GO:0043025 | CC | 137 | 38 | 17.74 | 0.00013 | neuronal cell body |
| GO:0070062 | CC | 778 | 135 | 100.72 | 0.00017 | extracellular vesicular exosome |
| GO:0032982 | CC | 15 | 8 | 1.94 | 0.00021 | myosin filament |
| GO:0005884 | CC | 53 | 19 | 6.86 | 0.00026 | actin filament |
| GO:0005829 | CC | 808 | 136 | 104.6 | 0.0003 | cytosol |
| GO:0045211 | CC | 184 | 41 | 23.82 | 0.0003 | postsynaptic membrane |
| GO:0030054 | CC | 653 | 129 | 84.54 | 0.00041 | cell junction |
| GO:0030424 | CC | 147 | 32 | 19.03 | 0.00047 | axon |
| GO:0016459 | CC | 141 | 39 | 18.25 | 0.00048 | myosin complex |
| GO:0005874 | CC | 279 | 62 | 36.12 | 0.00059 | microtubule |
| GO:0030315 | CC | 21 | 9 | 2.72 | 0.00068 | T-tubule |
| GO:0000145 | CC | 22 | 9 | 2.85 | 0.00102 | exocyst |
| GO:0016607 | CC | 50 | 15 | 6.47 | 0.0012 | nuclear speck |
| GO:0045121 | CC | 79 | 22 | 10.23 | 0.00147 | membrane raft |
| GO:0043204 | CC | 15 | 7 | 1.94 | 0.0015 | perikaryon |
| GO:0044297 | CC | 149 | 44 | 19.29 | 0.00202 | cell body |
| GO:0031143 | CC | 12 | 6 | 1.55 | 0.00215 | pseudopodium |
| GO:0044425 | CC | 4848 | 693 | 627.61 | 0.00218 | membrane part |
| GO:0014704 | CC | 33 | 12 | 4.27 | 0.00229 | intercalated disc |
| GO:0043197 | CC | 44 | 13 | 5.7 | 0.00291 | dendritic spine |
| GO:0005925 | CC | 145 | 31 | 18.77 | 0.00306 | focal adhesion |
| GO:0005859 | CC | 6 | 4 | 0.78 | 0.00338 | muscle myosin complex |
| GO:0005876 | CC | 36 | 11 | 4.66 | 0.00433 | spindle microtubule |
| GO:0030864 | CC | 34 | 11 | 4.4 | 0.00459 | cortical actin cytoskeleton |
| GO:0005654 | CC | 618 | 93 | 80 | 0.00479 | nucleoplasm |
| GO:0031982 | CC | 1081 | 176 | 139.94 | 0.005 | vesicle |
| GO:0005945 | CC | 10 | 5 | 1.29 | 0.00518 | 6-phosphofructokinase complex |
| GO:0000786 | CC | 42 | 12 | 5.44 | 0.00562 | nucleosome |
| GO:0016342 | CC | 59 | 15 | 7.64 | 0.00693 | catenin complex |
| GO:0043596 | CC | 18 | 5 | 2.33 | 0.00708 | nuclear replication fork |
| GO:0014069 | CC | 86 | 22 | 11.13 | 0.00859 | postsynaptic density |
| GO:0005911 | CC | 247 | 43 | 31.98 | 0.00865 | cell-cell junction |
| GO:0016529 | CC | 43 | 12 | 5.57 | 0.0088 | sarcoplasmic reticulum |

*BP: biological process; MF: molecular function; CC: cellular component

**Table S11.** Enriched Gene Ontology molecular function categories for unique genes in silver carp

| GO | Class* | Annotated | Significant | Expected | P-value | Term |
| --- | --- | --- | --- | --- | --- | --- |
| GO:0005001 | MF | 24 | 15 | 3.47 | 8.80E-08 | transmembrane receptor protein tyrosine phosphatase activity |
| GO:0005516 | MF | 110 | 34 | 15.92 | 8.20E-06 | calmodulin binding |
| GO:0003774 | MF | 231 | 54 | 33.44 | 1.20E-05 | motor activity |
| GO:0005515 | MF | 3931 | 690 | 569 | 2.00E-05 | protein binding |
| GO:0005509 | MF | 835 | 163 | 120.86 | 2.50E-05 | calcium ion binding |
| GO:0005525 | MF | 475 | 101 | 68.76 | 3.20E-05 | GTP binding |
| GO:0031748 | MF | 7 | 6 | 1.01 | 5.60E-05 | D1 dopamine receptor binding |
| GO:0004871 | MF | 1582 | 283 | 228.99 | 6.20E-05 | signal transducer activity |
| GO:0019871 | MF | 5 | 5 | 0.72 | 6.30E-05 | sodium channel inhibitor activity |
| GO:0017022 | MF | 31 | 14 | 4.49 | 0.00018 | myosin binding |
| GO:0008022 | MF | 60 | 20 | 8.68 | 0.00019 | protein C-terminus binding |
| GO:0044325 | MF | 52 | 18 | 7.53 | 0.00022 | ion channel binding |
| GO:0005216 | MF | 675 | 139 | 97.7 | 0.00024 | ion channel activity |
| GO:0016712 | MF | 18 | 9 | 2.61 | 0.00025 | oxidoreductase activity, acting on paired donors, with incorporation or reduction of molecular oxygen, reduced flavin or flavoprotein as one donor, and incorporation of one atom of oxygen |
| GO:0005488 | MF | 10873 | 1660 | 1573.84 | 0.00028 | binding |
| GO:0046982 | MF | 244 | 55 | 35.32 | 0.00044 | protein heterodimerization activity |
| GO:0003707 | MF | 115 | 30 | 16.65 | 0.00047 | steroid hormone receptor activity |
| GO:0097109 | MF | 9 | 6 | 1.3 | 0.00052 | neuroligin family protein binding |
| GO:0019901 | MF | 217 | 53 | 31.41 | 0.00082 | protein kinase binding |
| GO:0017166 | MF | 13 | 7 | 1.88 | 0.00101 | vinculin binding |
| GO:0030971 | MF | 20 | 9 | 2.89 | 0.00101 | receptor tyrosine kinase binding |
| GO:0003924 | MF | 264 | 57 | 38.21 | 0.00107 | GTPase activity |
| GO:0005088 | MF | 154 | 35 | 22.29 | 0.00111 | Ras guanyl-nucleotide exchange factor activity |
| GO:0032403 | MF | 488 | 96 | 70.64 | 0.00131 | protein complex binding |
| GO:0000166 | MF | 2999 | 515 | 434.1 | 0.00164 | nucleotide binding |
| GO:0042802 | MF | 426 | 93 | 61.66 | 0.00174 | identical protein binding |
| GO:0031432 | MF | 11 | 6 | 1.59 | 0.00219 | titin binding |
| GO:0015277 | MF | 11 | 6 | 1.59 | 0.00219 | kainate selective glutamate receptor activity |
| GO:0003708 | MF | 22 | 9 | 3.18 | 0.00229 | retinoic acid receptor activity |
| GO:0033130 | MF | 8 | 5 | 1.16 | 0.00242 | acetylcholine receptor binding |
| GO:0042803 | MF | 256 | 54 | 37.06 | 0.00244 | protein homodimerization activity |
| GO:0051015 | MF | 103 | 26 | 14.91 | 0.00271 | actin filament binding |
| GO:0005230 | MF | 169 | 45 | 24.46 | 0.00282 | extracellular ligand-gated ion channel activity |
| GO:0005432 | MF | 19 | 8 | 2.75 | 0.00323 | calcium:sodium antiporter activity |
| GO:0004890 | MF | 32 | 11 | 4.63 | 0.00391 | GABA-A receptor activity |
| GO:0002039 | MF | 28 | 10 | 4.05 | 0.00428 | p53 binding |
| GO:0001948 | MF | 33 | 12 | 4.78 | 0.00451 | glycoprotein binding |
| GO:0050998 | MF | 16 | 7 | 2.32 | 0.00454 | nitric-oxide synthase binding |
| GO:0035255 | MF | 16 | 7 | 2.32 | 0.00454 | ionotropic glutamate receptor binding |
| GO:0005254 | MF | 48 | 17 | 6.95 | 0.00465 | chloride channel activity |
| GO:0030306 | MF | 9 | 5 | 1.3 | 0.00479 | ADP-ribosylation factor binding |
| GO:0016594 | MF | 9 | 5 | 1.3 | 0.00479 | glycine binding |
| GO:0044323 | MF | 6 | 4 | 0.87 | 0.00514 | retinoic acid-responsive element binding |
| GO:0016934 | MF | 6 | 4 | 0.87 | 0.00514 | extracellular-glycine-gated chloride channel activity |
| GO:0022824 | MF | 6 | 4 | 0.87 | 0.00514 | transmitter-gated ion channel activity |
| GO:0019904 | MF | 294 | 62 | 42.56 | 0.00539 | protein domain specific binding |
| GO:0019905 | MF | 35 | 11 | 5.07 | 0.0057 | syntaxin binding |
| GO:0003779 | MF | 403 | 85 | 58.33 | 0.00572 | actin binding |
| GO:0005328 | MF | 38 | 12 | 5.5 | 0.00575 | neurotransmitter:sodium symporter activity |
| GO:0004712 | MF | 21 | 7 | 3.04 | 0.00628 | protein serine/threonine/tyrosine kinase activity |
| GO:0005234 | MF | 78 | 20 | 11.29 | 0.0066 | extracellular-glutamate-gated ion channel activity |
| GO:0050839 | MF | 62 | 18 | 8.97 | 0.00669 | cell adhesion molecule binding |
| GO:0097110 | MF | 22 | 8 | 3.18 | 0.00916 | scaffold protein binding |
| GO:0005249 | MF | 174 | 31 | 25.19 | 0.00917 | voltage-gated potassium channel activity |
| GO:0022857 | MF | 1287 | 244 | 186.29 | 0.0093 | transmembrane transporter activity |
| GO:0001968 | MF | 14 | 6 | 2.03 | 0.00966 | fibronectin binding |
| GO:0004887 | MF | 18 | 7 | 2.61 | 0.0097 | thyroid hormone receptor activity |
| GO:0048306 | MF | 18 | 7 | 2.61 | 0.0097 | calcium-dependent protein binding |
| GO:0005201 | MF | 55 | 15 | 7.96 | 0.0097 | extracellular matrix structural constituent |
| GO:0007156 | BP | 191 | 53 | 27.53 | 1.10E-06 | homophilic cell adhesion |
| GO:0034220 | BP | 1051 | 204 | 151.51 | 2.10E-06 | ion transmembrane transport |
| GO:0007185 | BP | 16 | 10 | 2.31 | 1.30E-05 | transmembrane receptor protein tyrosine phosphatase signaling pathway |
| GO:0030240 | BP | 23 | 12 | 3.32 | 2.20E-05 | skeletal muscle thin filament assembly |
| GO:0048169 | BP | 23 | 12 | 3.32 | 2.20E-05 | regulation of long-term neuronal synaptic plasticity |
| GO:0002026 | BP | 24 | 14 | 3.46 | 5.40E-05 | regulation of the force of heart contraction |
| GO:0014829 | BP | 7 | 6 | 1.01 | 5.50E-05 | vascular smooth muscle contraction |
| GO:0048011 | BP | 69 | 23 | 9.95 | 5.90E-05 | neurotrophin TRK receptor signaling pathway |
| GO:0086001 | BP | 26 | 13 | 3.75 | 6.10E-05 | cardiac muscle cell action potential |
| GO:0010881 | BP | 22 | 11 | 3.17 | 8.30E-05 | regulation of cardiac muscle contraction by regulation of the release of sequestered calcium ion |
| GO:0018105 | BP | 85 | 28 | 12.25 | 9.10E-05 | peptidyl-serine phosphorylation |
| GO:1902476 | BP | 45 | 17 | 6.49 | 9.30E-05 | chloride transmembrane transport |
| GO:0030168 | BP | 85 | 25 | 12.25 | 0.0001 | platelet activation |
| GO:0001757 | BP | 26 | 12 | 3.75 | 0.00011 | somite specification |
| GO:0001501 | BP | 453 | 75 | 65.3 | 0.00011 | skeletal system development |
| GO:0010976 | BP | 44 | 17 | 6.34 | 0.00012 | positive regulation of neuron projection development |
| GO:0030007 | BP | 13 | 8 | 1.87 | 0.00012 | cellular potassium ion homeostasis |
| GO:0071625 | BP | 20 | 10 | 2.88 | 0.00018 | vocalization behavior |
| GO:0006987 | BP | 8 | 6 | 1.15 | 0.00019 | activation of signaling protein activity involved in unfolded protein response |
| GO:0031444 | BP | 8 | 6 | 1.15 | 0.00019 | slow-twitch skeletal muscle fiber contraction |
| GO:2000650 | BP | 8 | 6 | 1.15 | 0.00019 | negative regulation of sodium ion transmembrane transporter activity |
| GO:0007612 | BP | 64 | 23 | 9.23 | 0.00024 | learning |
| GO:0051481 | BP | 11 | 7 | 1.59 | 0.00025 | negative regulation of cytosolic calcium ion concentration |
| GO:0072578 | BP | 11 | 7 | 1.59 | 0.00025 | neurotransmitter-gated ion channel clustering |
| GO:0001941 | BP | 11 | 7 | 1.59 | 0.00025 | postsynaptic membrane organization |
| GO:0038095 | BP | 36 | 14 | 5.19 | 0.00027 | Fc-epsilon receptor signaling pathway |
| GO:0007411 | BP | 327 | 77 | 47.14 | 0.00032 | axon guidance |
| GO:0048014 | BP | 6 | 5 | 0.86 | 0.00033 | Tie signaling pathway |
| GO:0051336 | BP | 829 | 150 | 119.5 | 0.00041 | regulation of hydrolase activity |
| GO:0071353 | BP | 9 | 6 | 1.3 | 0.00051 | cellular response to interleukin-4 |
| GO:0035022 | BP | 9 | 6 | 1.3 | 0.00051 | positive regulation of Rac protein signal transduction |
| GO:0048791 | BP | 9 | 6 | 1.3 | 0.00051 | calcium ion-dependent exocytosis of neurotransmitter |
| GO:0060333 | BP | 9 | 6 | 1.3 | 0.00051 | interferon-gamma-mediated signaling pathway |
| GO:0031345 | BP | 78 | 15 | 11.24 | 0.00051 | negative regulation of cell projection organization |
| GO:0043401 | BP | 119 | 31 | 17.15 | 0.0006 | steroid hormone mediated signaling pathway |
| GO:0055003 | BP | 36 | 15 | 5.19 | 0.00061 | cardiac myofibril assembly |
| GO:0065007 | BP | 8696 | 1320 | 1253.57 | 0.00064 | biological regulation |
| GO:0016477 | BP | 791 | 139 | 114.03 | 0.00066 | cell migration |
| GO:0048741 | BP | 80 | 22 | 11.53 | 0.00095 | skeletal muscle fiber development |
| GO:0071688 | BP | 15 | 8 | 2.16 | 0.00101 | striated muscle myosin thick filament assembly |
| GO:0006813 | BP | 265 | 52 | 38.2 | 0.00108 | potassium ion transport |
| GO:0035176 | BP | 28 | 11 | 4.04 | 0.00109 | social behavior |
| GO:0010765 | BP | 10 | 6 | 1.44 | 0.00111 | positive regulation of sodium ion transport |
| GO:0071313 | BP | 10 | 6 | 1.44 | 0.00111 | cellular response to caffeine |
| GO:0007214 | BP | 41 | 14 | 5.91 | 0.00123 | gamma-aminobutyric acid signaling pathway |
| GO:0032469 | BP | 10 | 5 | 1.44 | 0.00191 | endoplasmic reticulum calcium ion homeostasis |
| GO:0071514 | BP | 11 | 5 | 1.59 | 0.00191 | genetic imprinting |
| GO:0006983 | BP | 5 | 4 | 0.72 | 0.00191 | ER overload response |
| GO:0061049 | BP | 5 | 4 | 0.72 | 0.00191 | cell growth involved in cardiac muscle cell development |
| GO:0030049 | BP | 5 | 4 | 0.72 | 0.00191 | muscle filament sliding |
| GO:0098901 | BP | 5 | 4 | 0.72 | 0.00191 | regulation of cardiac muscle cell action potential |
| GO:0032856 | BP | 12 | 5 | 1.73 | 0.00191 | activation of Ras GTPase activity |
| GO:0031032 | BP | 144 | 48 | 20.76 | 0.00196 | actomyosin structure organization |
| GO:0016055 | BP | 361 | 55 | 52.04 | 0.00201 | Wnt signaling pathway |
| GO:0060048 | BP | 67 | 25 | 9.66 | 0.00203 | cardiac muscle contraction |
| GO:0045010 | BP | 42 | 14 | 6.05 | 0.00213 | actin nucleation |
| GO:0060997 | BP | 18 | 8 | 2.59 | 0.00214 | dendritic spine morphogenesis |
| GO:0032793 | BP | 11 | 6 | 1.59 | 0.00214 | positive regulation of CREB transcription factor activity |
| GO:0006883 | BP | 11 | 6 | 1.59 | 0.00214 | cellular sodium ion homeostasis |
| GO:0071456 | BP | 31 | 11 | 4.47 | 0.00222 | cellular response to hypoxia |
| GO:0007173 | BP | 66 | 22 | 9.51 | 0.00228 | epidermal growth factor receptor signaling pathway |
| GO:0045184 | BP | 812 | 129 | 117.05 | 0.00233 | establishment of protein localization |
| GO:0035235 | BP | 87 | 23 | 12.54 | 0.00234 | ionotropic glutamate receptor signaling pathway |
| GO:0007191 | BP | 8 | 5 | 1.15 | 0.00237 | adenylate cyclase-activating dopamine receptor signaling pathway |
| GO:0031532 | BP | 40 | 14 | 5.77 | 0.00258 | actin cytoskeleton reorganization |
| GO:0071277 | BP | 15 | 7 | 2.16 | 0.00284 | cellular response to calcium ion |
| GO:0007158 | BP | 15 | 7 | 2.16 | 0.00284 | neuron cell-cell adhesion |
| GO:0008045 | BP | 49 | 15 | 7.06 | 0.00287 | motor neuron axon guidance |
| GO:0071436 | BP | 8 | 5 | 1.15 | 0.00299 | sodium ion export |
| GO:0044557 | BP | 12 | 5 | 1.73 | 0.00299 | relaxation of smooth muscle |
| GO:0031122 | BP | 19 | 8 | 2.74 | 0.00315 | cytoplasmic microtubule organization |
| GO:0009790 | BP | 1572 | 257 | 226.61 | 0.00347 | embryo development |
| GO:0043170 | BP | 6644 | 896 | 957.77 | 0.00371 | macromolecule metabolic process |
| GO:0086064 | BP | 24 | 11 | 3.46 | 0.00374 | cell communication by electrical coupling involved in cardiac conduction |
| GO:0007628 | BP | 12 | 6 | 1.73 | 0.00377 | adult walking behavior |
| GO:0007229 | BP | 85 | 22 | 12.25 | 0.00381 | integrin-mediated signaling pathway |
| GO:0009069 | BP | 665 | 121 | 95.86 | 0.00439 | serine family amino acid metabolic process |
| GO:0008344 | BP | 32 | 14 | 4.61 | 0.00454 | adult locomotory behavior |
| GO:0048167 | BP | 68 | 26 | 9.8 | 0.00459 | regulation of synaptic plasticity |
| GO:0055119 | BP | 18 | 10 | 2.59 | 0.00467 | relaxation of cardiac muscle |
| GO:2000300 | BP | 9 | 5 | 1.3 | 0.0047 | regulation of synaptic vesicle exocytosis |
| GO:0097120 | BP | 9 | 5 | 1.3 | 0.0047 | receptor localization to synapse |
| GO:0022617 | BP | 9 | 5 | 1.3 | 0.0047 | extracellular matrix disassembly |
| GO:1901897 | BP | 9 | 5 | 1.3 | 0.0047 | regulation of relaxation of cardiac muscle |
| GO:0051965 | BP | 33 | 11 | 4.76 | 0.00495 | positive regulation of synapse assembly |
| GO:0035994 | BP | 6 | 4 | 0.86 | 0.00506 | response to muscle stretch |
| GO:0033206 | BP | 6 | 4 | 0.86 | 0.00506 | meiotic cytokinesis |
| GO:0016344 | BP | 6 | 4 | 0.86 | 0.00506 | meiotic chromosome movement towards spindle pole |
| GO:0032852 | BP | 6 | 4 | 0.86 | 0.00506 | positive regulation of Ral GTPase activity |
| GO:0098735 | BP | 6 | 4 | 0.86 | 0.00506 | positive regulation of the force of heart contraction |
| GO:0006782 | BP | 6 | 4 | 0.86 | 0.00506 | protoporphyrinogen IX biosynthetic process |
| GO:0042953 | BP | 6 | 4 | 0.86 | 0.00506 | lipoprotein transport |
| GO:0031063 | BP | 18 | 8 | 2.59 | 0.00613 | regulation of histone deacetylation |
| GO:0021514 | BP | 34 | 10 | 4.9 | 0.00649 | ventral spinal cord interneuron differentiation |
| GO:0086004 | BP | 29 | 10 | 4.18 | 0.00659 | regulation of cardiac muscle cell contraction |
| GO:0045087 | BP | 174 | 40 | 25.08 | 0.00794 | innate immune response |
| GO:0051289 | BP | 26 | 9 | 3.75 | 0.00814 | protein homotetramerization |
| GO:0035264 | BP | 54 | 13 | 7.78 | 0.00814 | multicellular organism growth |
| GO:0050806 | BP | 37 | 13 | 5.33 | 0.00823 | positive regulation of synaptic transmission |
| GO:0006461 | BP | 610 | 110 | 87.93 | 0.00824 | protein complex assembly |
| GO:0060314 | BP | 24 | 10 | 3.46 | 0.00825 | regulation of ryanodine-sensitive calcium-release channel activity |
| GO:0048041 | BP | 34 | 11 | 4.9 | 0.00826 | focal adhesion assembly |
| GO:0010613 | BP | 10 | 5 | 1.44 | 0.0083 | positive regulation of cardiac muscle hypertrophy |
| GO:0051968 | BP | 10 | 5 | 1.44 | 0.0083 | positive regulation of synaptic transmission, glutamatergic |
| GO:0001892 | BP | 18 | 6 | 2.59 | 0.0083 | embryonic placenta development |
| GO:0046777 | BP | 107 | 26 | 15.42 | 0.00839 | protein autophosphorylation |
| GO:0007268 | BP | 553 | 133 | 79.72 | 0.00844 | synaptic transmission |
| GO:0002027 | BP | 45 | 14 | 6.49 | 0.00888 | regulation of heart rate |
| GO:0001525 | BP | 374 | 63 | 53.91 | 0.00893 | angiogenesis |
| GO:0046339 | BP | 45 | 13 | 6.49 | 0.00909 | diacylglycerol metabolic process |
| GO:0008217 | BP | 63 | 16 | 9.08 | 0.00931 | regulation of blood pressure |
| GO:0055085 | BP | 1464 | 277 | 211.04 | 0.00932 | transmembrane transport |
| GO:0042059 | BP | 14 | 6 | 2.02 | 0.00947 | negative regulation of epidermal growth factor receptor signaling pathway |
| GO:0048755 | BP | 14 | 6 | 2.02 | 0.00947 | branching morphogenesis of a nerve |
| GO:0090129 | BP | 14 | 6 | 2.02 | 0.00947 | positive regulation of synapse maturation |
| GO:0007202 | BP | 18 | 7 | 2.59 | 0.00949 | activation of phospholipase C activity |
| GO:0006184 | BP | 849 | 160 | 122.39 | 0.00963 | GTP catabolic process |
| GO:0030054 | CC | 699 | 170 | 101 | 9.10E-09 | cell junction |
| GO:0005886 | CC | 2490 | 501 | 359.77 | 6.10E-07 | plasma membrane |
| GO:0030666 | CC | 35 | 16 | 5.06 | 6.30E-07 | endocytic vesicle membrane |
| GO:0016459 | CC | 145 | 49 | 20.95 | 2.00E-06 | myosin complex |
| GO:0016020 | CC | 6820 | 1100 | 985.4 | 2.40E-06 | membrane |
| GO:0005829 | CC | 837 | 159 | 120.94 | 2.90E-06 | cytosol |
| GO:0005925 | CC | 162 | 46 | 23.41 | 3.00E-06 | focal adhesion |
| GO:0070062 | CC | 806 | 162 | 116.46 | 4.00E-06 | extracellular vesicular exosome |
| GO:0045211 | CC | 184 | 50 | 26.59 | 4.50E-06 | postsynaptic membrane |
| GO:0043005 | CC | 427 | 119 | 61.7 | 4.60E-06 | neuron projection |
| GO:0032982 | CC | 13 | 9 | 1.88 | 1.10E-05 | myosin filament |
| GO:0030315 | CC | 23 | 12 | 3.32 | 2.30E-05 | T-tubule |
| GO:0042734 | CC | 47 | 18 | 6.79 | 4.80E-05 | presynaptic membrane |
| GO:0005911 | CC | 250 | 51 | 36.12 | 6.50E-05 | cell-cell junction |
| GO:0014069 | CC | 96 | 30 | 13.87 | 7.60E-05 | postsynaptic density |
| GO:0005884 | CC | 56 | 22 | 8.09 | 9.40E-05 | actin filament |
| GO:0008305 | CC | 58 | 20 | 8.38 | 0.00011 | integrin complex |
| GO:0031234 | CC | 79 | 23 | 11.41 | 0.00017 | extrinsic component of cytoplasmic side of plasma membrane |
| GO:0032839 | CC | 8 | 6 | 1.16 | 0.00019 | dendrite cytoplasm |
| GO:0044306 | CC | 45 | 15 | 6.5 | 0.00033 | neuron projection terminus |
| GO:0042643 | CC | 6 | 5 | 0.87 | 0.00033 | actomyosin, actin portion |
| GO:0031105 | CC | 25 | 11 | 3.61 | 0.00035 | septin complex |
| GO:0048471 | CC | 199 | 47 | 28.75 | 0.00036 | perinuclear region of cytoplasm |
| GO:0034707 | CC | 24 | 13 | 3.47 | 0.00038 | chloride channel complex |
| GO:0030496 | CC | 73 | 22 | 10.55 | 0.00044 | midbody |
| GO:0072562 | CC | 15 | 8 | 2.17 | 0.00047 | blood microparticle |
| GO:0045202 | CC | 536 | 134 | 77.45 | 0.00053 | synapse |
| GO:0014704 | CC | 32 | 13 | 4.62 | 0.00073 | intercalated disc |
| GO:0044224 | CC | 13 | 7 | 1.88 | 0.001 | juxtaparanode region of axon |
| GO:0030425 | CC | 205 | 55 | 29.62 | 0.00119 | dendrite |
| GO:0001725 | CC | 33 | 12 | 4.77 | 0.00148 | stress fiber |
| GO:0016323 | CC | 72 | 20 | 10.4 | 0.00159 | basolateral plasma membrane |
| GO:0005654 | CC | 637 | 104 | 92.04 | 0.00164 | nucleoplasm |
| GO:0031674 | CC | 82 | 26 | 11.85 | 0.0017 | I band |
| GO:0032154 | CC | 38 | 13 | 5.49 | 0.00182 | cleavage furrow |
| GO:0005913 | CC | 32 | 12 | 4.62 | 0.0021 | cell-cell adherens junction |
| GO:0034704 | CC | 115 | 27 | 16.62 | 0.00221 | calcium channel complex |
| GO:0030018 | CC | 68 | 19 | 9.83 | 0.00282 | Z disc |
| GO:0055038 | CC | 23 | 9 | 3.32 | 0.00323 | recycling endosome membrane |
| GO:0045121 | CC | 69 | 21 | 9.97 | 0.00481 | membrane raft |
| GO:0005887 | CC | 784 | 162 | 113.28 | 0.00483 | integral component of plasma membrane |
| GO:0032281 | CC | 42 | 13 | 6.07 | 0.0049 | alpha-amino-3-hydroxy-5-methyl-4-isoxazolepropionic acid selective glutamate receptor complex |
| GO:0043025 | CC | 145 | 36 | 20.95 | 0.00499 | neuronal cell body |
| GO:0016935 | CC | 6 | 4 | 0.87 | 0.00511 | glycine-gated chloride channel complex |
| GO:0032983 | CC | 21 | 8 | 3.03 | 0.00658 | kainate selective glutamate receptor complex |
| GO:0008180 | CC | 17 | 7 | 2.46 | 0.00669 | COP9 signalosome |
| GO:0005912 | CC | 202 | 60 | 29.19 | 0.0089 | adherens junction |
| GO:0031526 | CC | 22 | 8 | 3.18 | 0.00905 | brush border membrane |
| GO:0030424 | CC | 154 | 42 | 22.25 | 0.00971 | axon |

*BP: biological process; MF: molecular function; CC: cellular component

Table S12. Functional prediction of nonsynonymous SNP in two F1 hybrid individuals (H1 and H2) via Polyphen-2e

| name | type | db | No of SNPs |
| --- | --- | --- | --- |
| H1 | benign | HumanDiv | 8365 |
| H1 | benign | HumanVar | 9297 |
| H1 | possibly_damaging | HumanDiv | 1551 |
| H1 | possibly_damaging | HumanVar | 1005 |
| H1 | probably_damaging | HumanDiv | 821 |
| H1 | probably_damaging | HumanVar | 435 |
| H2 | benign | HumanDiv | 9349 |
| H2 | benign | HumanVar | 10398 |
| H2 | possibly_damaging | HumanDiv | 1768 |
| H2 | possibly_damaging | HumanVar | 1155 |
| H2 | probably_damaging | HumanDiv | 899 |
| H2 | probably_damaging | HumanVar | 463 |

Table S13. Functional prediction of nonsynonymous SNP in two F1 hybrid individuals (H1 and H2) via SIFT

|  | Type | #SNP count |
| --- | --- | --- |
| H1 | Deleterious | 9023 |
| H1 | Tolerated | 45002 |
| H1 | Not scored | 10614 |
| H1 | Total SNP locals | 74651 |
| H1 | Predicted SNP locals | 64639 |
| H1 | Not Predicted locals | 10012 |
| H2 | Deleterious | 8969 |
| H2 | Tolerated | 44743 |
| H2 | Tolerated | 10539 |
| H2 | Total SNP locals | 75354 |
| H2 | Predicted SNP locals | 64251 |
| H2 | Not Predicted locals | 11103 |

Table S14. Fertilization rate, hatch rate and deformation rate of intra- and interspecific crosses of bighead carp (BC) and silver carp (SC), with the number of samples indicated in parentheses

|  | BC×BC | SC×SC | BC×SC | SC×BC |
| --- | --- | --- | --- | --- |
| Fertilization rate | 0.911±0.143 (39) | 0.932±0.701(43) | 0.953±0.036 (27) | 0.926±0.889 (30) |
| Hatch rate | 0.982±0.200 (16) | 0.965±0.277 (10) | 0.995±0.009 (10) | 0.984±0.269 (11) |
| Deformation rate | 0.013±0.029 (19) | 0.007±0.011 (23) | 0.012±0.023 (23) | 0.023±0.026 (22) |

**References**

1. Howe K, et al. (2013) The zebrafish reference genome sequence and its relationship to the human genome. Nature 496(7446):498-503.

2. Xu P, et al. (2014) Genome sequence and genetic diversity of the common carp, Cyprinus carpio. Nat Genet 46(11):1212-1219.

3. McGaugh SE, et al. (2014) The cavefish genome reveals candidate genes for eye loss. Nat Commun 5.
